# Polymer‐Driven Co‐Assembly of Achiral and Chiral Nanoparticles into Plasmonic Nanoclusters with Quantitatively Modulated Optical Chirality

**DOI:** 10.1002/advs.202504850

**Published:** 2025-06-19

**Authors:** Chongyang Yao, Huibin He, Weijia Kong, Liangyu Hu, Xiaoxue Shen, Jing Tao, Yutao Sang, Zhihong Nie

**Affiliations:** ^1^ State Key Laboratory of Molecular Engineering of Polymers State Key Laboratory of Surface Physics and Key Laboratory of Micro and Nano Photonic Structures (Ministry of Education) Fudan University Shanghai 200438 P. R. China

**Keywords:** chiral nanoparticles, hotspots, inherent plasmonic chirality, plasmonic nanoclusters, polymer‐driven self‐assembly

## Abstract

Chiral plasmonic nanoassemblies demonstrate enhanced chiral optical activity through plasmonic mode coupling, holding transformative potential for applications in sensing, catalysis, and quantum‐optical technologies. However, the mechanisms underlying this enhancement—particularly the roles of structural geometry, plasmonic coupling, and chiral field amplification—remain incompletely elucidated. A significant challenge persists in designing coupled nanoassemblies with precisely controlled nanostructures to systematically investigate chirality enhancement. Departing from conventional approaches that incorporate chiral molecules, we present the co‐assembly of achiral and chiral plasmonic nanoparticles (NPs) into **AB_n_**‐type nanoclustersand the correlation between inherent plasmonic chirality and the quantity of hotspots. Complementary polymer‐grafted achiral nanospheres and chiral nano arrows assemble into stable **AB_n_** clusters through a combination of electrostatic interactions and hydrogen bonding. The coordination number (***n***) of **AB_n_** can be tuned from 2 to 7 by adjusting polymer configurations through modulation of solution pH. The *g*‐factor of **AB_n_** exhibits a linear increase with the ***n*** value of **AB_n_**. Simulation results indicate that the enhanced optical chirality arises from the increase in electric field strength due to the increasing number of hotspots within the NP assemblies.

## Graphical abstract

This work describes the polymer‐mediated co‐assembly of chiral gold nanoarrows and achiral nanospheres into **AB_n_** plasmonic clusters enabling precise control over coordination number (***n *** = 2–7) via pH modulation. Experimental and simulation results reveal a linear enhancement of optical chirality (*g*‐factor) with increasing ***n***, attributed to electric field amplification at interparticle hotspots. This quantitative approach advances programmable chiral nanostructures.

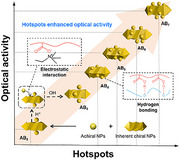

## 1 Introduction

**Figure 1:**
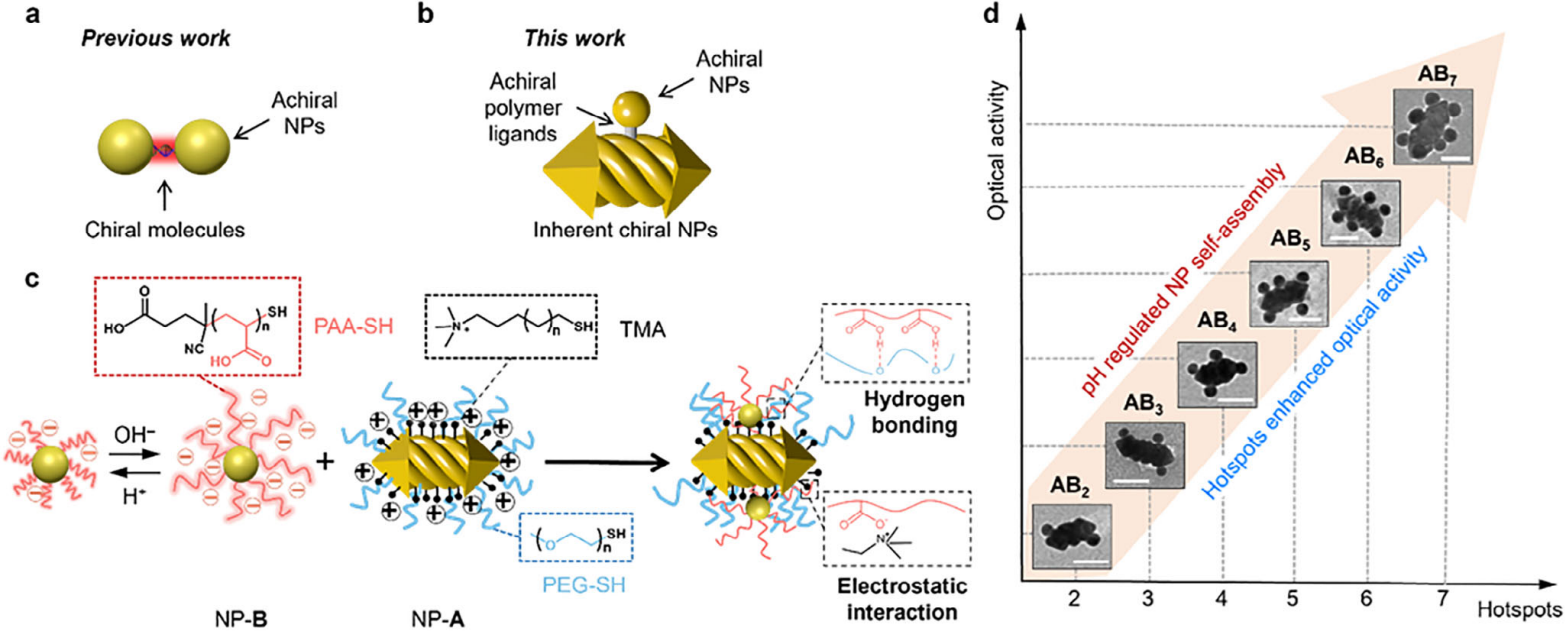
Schematic representation of polymer‐grated NPs and their assemblies showcasing controlled hotspots and enhanced optical activity. Representation of the hotspot model from a) previous research and b) the current study. c) Molecular structures of the polymer ligands and schematic illustration of the hydrogen bonding and electrostatic interactions‐mediated binary NPs self‐assembly. d) Schematic illustration of a linear correlation between inherent optical activity intensities and the quantity of hotspots. Inserts are transmission electron microscope (TEM) images of the **AB_n_** clusters with different ***n*** values. Scale bars are 50 nm.

Chiral plasmonic materials have garnered significant attention over the last decade, due to their distinctive opto‐photonic properties and broad potential applications such as asymmetric catalysis^[^ ^1^, ^2^, ^3^ ^]^, 3D displays,^[^ ^4^ ^]^ information security,^[^ ^5^ ^]^ biotherapeutics,^[^ ^6^, ^7^ ^]^ and so forth.^[^ ^8^ ^]^ In particular, when plasmonic nanoparticles (NPs) are arranged at close distances, the plasmonic coupling between neighboring NPs creates so‐called hotspots characterized by an intensified optical electric field at the junctions of NPs.^[^ ^9^, ^10^, ^11^ ^]^ The strength of hotspots increases as the interparticle distance is decreased. These hotspots can effectively enhance spectroscopic signals, such as surface‐enhanced Raman scattering,^[^ ^12^, ^13^, ^14^ ^]^ fluorescence,^[^ ^15^, ^16^ ^]^ Fano resonances,^[^ ^17^, ^18^ ^]^ and optical circular dichroism (CD).^[^ ^19^, ^20^, ^21^ ^]^ Theoretical studies^[^ ^22^, ^23^ ^]^ and experimental results^[^ ^24^, ^25^, ^26^ ^]^ have shown that when a chiral molecule is in close contact with a metal surface, it can transfer the CD signal in the ultraviolet range into the frequency domain of the equipartitioned exciton. Specially, when chiral molecules are placed within hotspots of plasmonic nanostructures (e.g., NP dimers), the CD signals of the molecule/NP complexes are significantly amplified at the characteristic absorption wavelength of localized surface plasmon resonances (LSPR) (**Figure** **1**a).^[^ ^27^, ^28^, ^29^, ^30^ ^]^

Clusters of NPs in close proximity are typically fabricated through intermolecular forces‐driven assembly of NPs in solution phases. With respect to chiral plasmonic nanoclusters,^[^ ^20^, ^31^, ^32^, ^33^ ^]^ chiral biomolecules such as amino acids,^[^ ^27^, ^28^ ^]^ peptides,^[^ ^7^, ^29^, ^30^ ^]^ proteins,^[^ ^34^, ^35^, ^36^ ^]^ and DNA^[^ ^37^, ^38^, ^39^, ^40^, ^41^ ^]^ are used to induce the chiral organization of NPs. Typically, DNA often offers high precision in programming the spatial arrangement and interparticle junctions of NPs in chiral clusters with different configurations.^[^ ^42^, ^43^ ^]^ For instance, the precise positioning of four distinct‐sized gold NPs (AuNPs) at the tetrahedral vertices of DNA scaffolds generated rigid chiral plasmonic pyramids.^[^ ^44^ ^]^ This approach was extended to create heterogeneous chiral pyramids containing AuNPs, silver NPs, and quantum dots.^[^ ^45^ ^]^ DNA origami nanostructures (comprising a 24‐DNA helix bundle) with pre‐designed binding sites were used to direct the precise helical arrangement of AuNPs to form 3D chiral structures.^[^ ^41^ ^]^ More recently, human islet amyloid polypeptides (hIAPPs) were covalently anchored to the surface of gold nanorods capped with cetyltrimethylammonium bromide bilayers. The supramolecular self‐assembly of hIAPP into β‐sheet fibrils drove the end‐to‐end association of nanorods into left‐handed helices with a long‐range registry, amplifying the chiral signals by over 4600‐fold compared to isolated nanorod‐peptide conjugates.^[^ ^7^ ^]^ The observed chirality in these NP assemblies primarily stems from the spatial arrangement of achiral NPs without structural chirality. Nevertheless, plasmonic nanoclusters involving chiral NPs and their optical responses associated with inherent plasmonic chirality remain unexplored at present (Figure 1b).

Herein, we report the complementary polymer‐directed assembly of chiral/achiral NPs into clusters with controlled coordination numbers and the correlation between inherent plasmonic chirality and the quantity of hotspots. Chiral plasmonic NPs, namely NP‐**A**s, comprise helically grooved gold nanoarrows grafted with positively charged (11‐mercaptoundecyl)‐*N,N,N*‐trimethylammonium bromide (TMA) and polyethylene glycol (PEG) (Figure 1c). PEG ligands provide a steric effect in stabilizing NPs and serve as hydrogen bonding acceptors during the self‐assembly. Achiral plasmonic NPs, namely NP‐**B**s, consist of achiral AuNPs functionalized with negatively charged polyacrylic acid (PAA) (Figure 1d). The electrostatic interaction between TMA and PAA coupled with hydrogen‐bonding interactions between PAA and PEG drive the binary NPs to assemble into **AB_n_** clusters in an aqueous solution (***n*** is the coordination number of NP‐**B**s). The ***n*** value of **AB_n_** can be precisely controlled from 2 to 7 by modulating the repeated cycles of pH variations. We demonstrate that the optical chirality enhances linearly with increasing the number of absorbed achiral NP‐**B**s, that is, the number of hotspots within the NP clusters. Our simulation indicates that the overall strength of the local electric field within individual nanoclusters is linearly correlated to the number of hotspots, which quantitatively determines the chiral optical response. This work bridges the gap between nanoscale geometric engineering with macroscopic optical chirality, enabling the design of advanced chiral materials for applications in ultrasensitive chiral sensing, asymmetric catalysis, and quantum‐optical devices.

## 2 Results and Discussion

**Figure 2:**
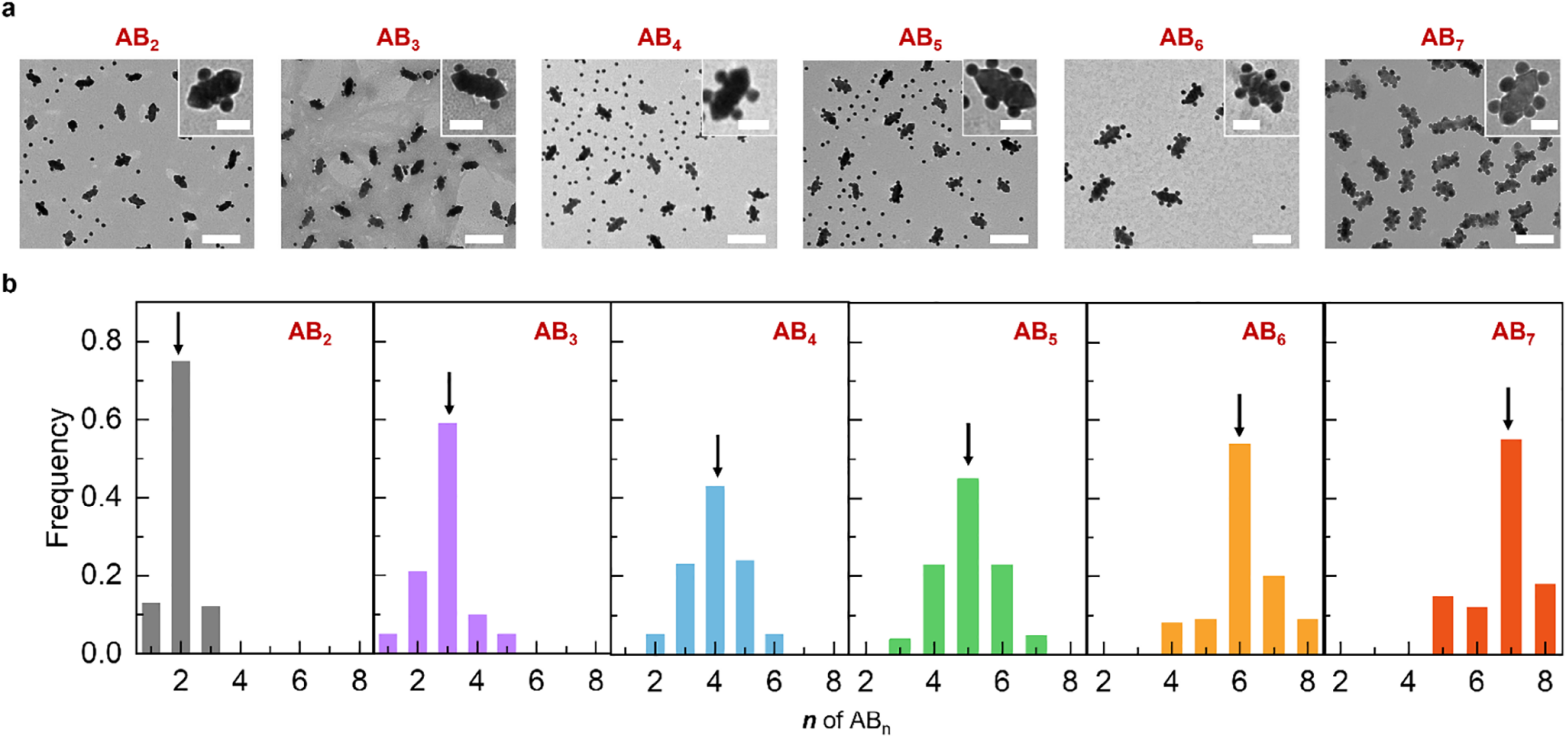
Structure of the NP assemblies. a) TEM images of **AB_2_**, **AB_3_**, **AB_4_**, **AB_5_**, **AB_6_**, and **AB_7_** clusters. b) The corresponding distribution of the **AB_n_** clusters. Scale bars are 200 nm and inset scale bars are 50 nm.

The helical grooved nanoarrows (NP‐**A**s) were selected as the chiral component and synthesized by adding *L*‐ or *D*‐cysteine as the chiral inducer into the growth solution of gold nanorods (see Figure S1, Supporting Information, and Experimental Section).^[^ ^46^ ^]^ The achiral component is Au nanospheres (NP‐**B**s) with a diameter of 15.1 ± 1.2 nm (Figure S2, Supporting Information).^[^ ^47^, ^48^, ^49^, ^50^ ^]^ NP‐**A**s and NP‐**B**s were functionalized with complementary polymers through a ligand exchange process and subsequently dispersed in water. Specifically, the negatively charged PAA capable of hydrogen bond donation and positively charged TMA were grafted on NP‐**A**s and NP‐**B**s, respectively (Figure 1). The synthesis route of PAA is shown in Figure S3 (Supporting Information). Additionally, NP‐**A**s were functionalized with PEG as hydrogen‐bond acceptors to precisely modulate interparticle interactions. When dispersions of NP‐**Bs** and NP‐**A**s were mixed, electrostatic attraction between negatively charged PAA on NP‐**A**s and positively charged TMA on NP‐**B**s, along with hydrogen‐bonding between PAA's carboxyl groups (‐COOH) and PEG's ether oxygen atoms (─O─), trigger the assembly of these NPs to form **AB_2_** clusters at a yield of ≈75% (**Figure** **2**a; Figure S4, Supporting Information). The achiral NP‐**B**s are mainly absorbed on the side of nanoarrows, thus resulting in a redshift from 708 to 738 nm in the peak wavelength of the longitudinal LSPR of the nanoarrows (Figure S5a, Supporting Information).

The as‐formed **AB_2_** clusters maintain stability in neutral and base aqueous solutions for several months due to the spatial reinforcement provided by polymer ligands and the substantial negative charge conferred by the PAA (Figure S6, Supporting Information). Interestingly, thanks to the pH responsiveness of the inter‐particle forces, the **AB_2_** continued to react with the free NP‐**B**s and transited to **AB_3_** with a yield of 59% after the pH of the solution was decreased from 10.0 to 3.0 and then resumed back to 10.0 (Figure S7, Supporting Information). By controlling the cycles of repeating pH variations, **AB_n_** clusters (***n *** = 4–7) with varying numbers of NP‐**B**s were further constructed (see a detailed discussion of the mechanism in the next section). The yields of **AB_4_**, **AB_5_**, **AB_6,_** and **AB_7_** clusters were 43%, 45%, 54%, and 54%, respectively (Figure 2b). The TEM micrographs revealed that the NP‐**B**s adsorbed on the flanks of the gold nanoarrows were predominantly localized in the intermediate regions between the two helical grooves. This spatial preference can be attributed to the geometric matching, given that the pitch dimension of the NP‐**A**s measures 16 nm while the characteristic size of the NP‐**B**s is 15 nm. High‐contrast TEM images revealed that the achiral NP‐**B**s are randomly assembled around the chiral NP‐**A**s rather than forming spiral or helical arrangements (Figure S8, Supporting Information). This is primarily attributed to the nondirectional and strong hydrogen bonding and electrostatic interactions mediated by the complementary copolymer ligands. A further increase in the pH cycles resulted in the association of the clusters to form large aggregates (Figure S9, Supporting Information).

**Figure 3:**
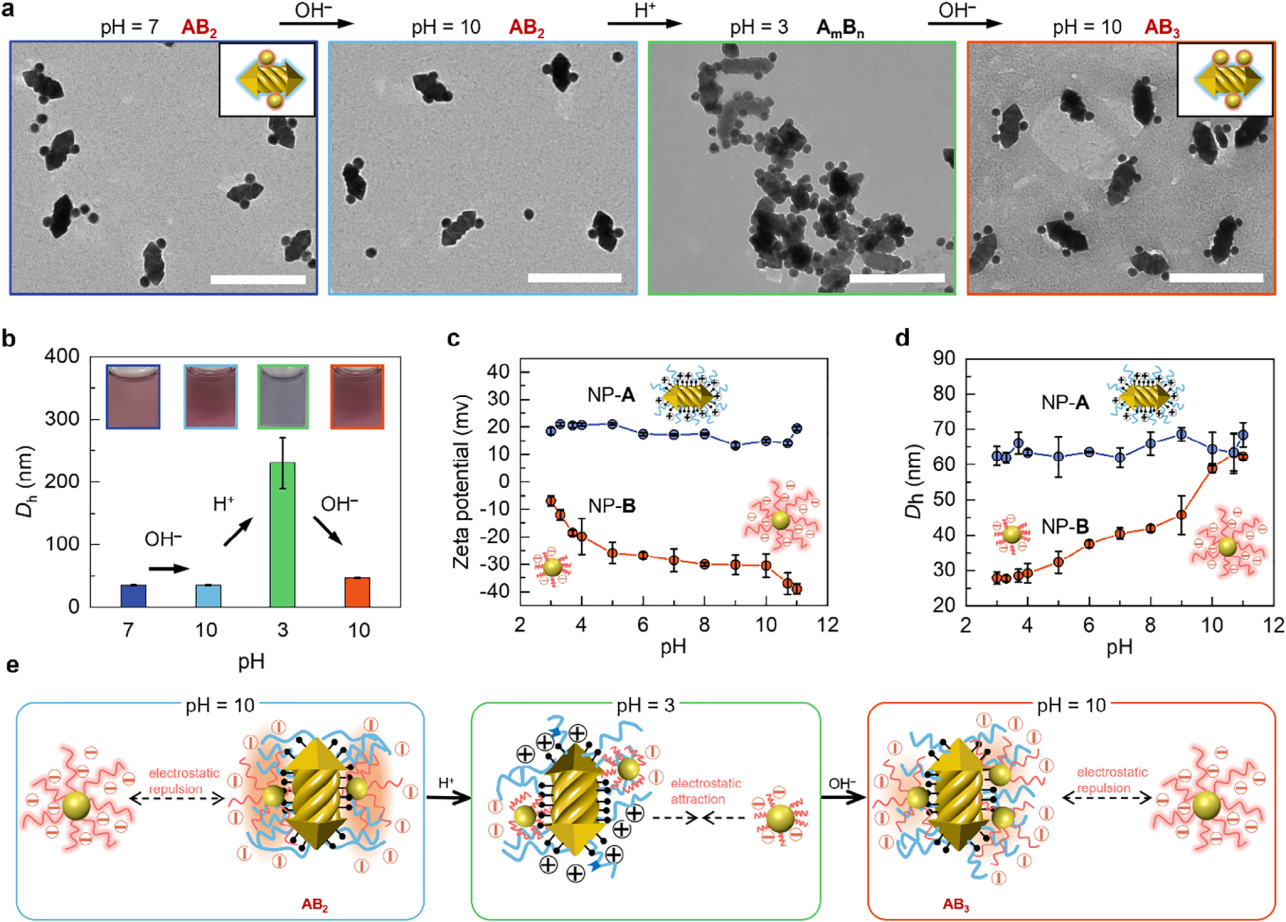
TEM images and Schematic diagram of **AB_3_** cluster formation. a) TEM images and b) *D*_h_ of the NP clusters assembled at different pH values. Inserts in (b) are photos of the NP solutions under different pH values. c) zeta potential values and d) *D*_h_ of NP‐**A**s and NP‐**B**s at different pH values. Insets are schematic diagrams of NP‐**A**s and NP‐**B**s. e) Schematic illustration of structure evolution as the pH changed from 10.0 to 3.0 and back to 10.0. Scale bars are 200 nm.

The presence of PAA ligands not only provides the negative charge and hydrogen bonding for the self‐assembly but also impacts pH‐responsive stimulus capabilities to the NPs. We monitored the structural evolution of NP assemblies throughout the pH cycle using TEM imaging, dynamic light scattering (DLS), and zeta potential measurements (**Figure** **3**a–d). Under acidic conditions (e.g., pH 3.0), **AB_2_** further assembly into large aggregates (denoted as **A_m_B_n_**), coinciding with a shift in solution color from red to gray (Figure 3a and insert in 3b). Details regarding changes in **AB_2_** clusters as pH decreases are given in Figures S10–S12 (Supporting Information). If the solution is maintained in an acidic state such as pH 3.0 for a period of time, the size of the aggregates increases and the UV/Vis absorption spectrum is red‐shifted (Figure S13, Supporting Information). Upon increasing the pH from 3.0 to 10.0, the aggregated NPs disassembled to reform **AB_n_** clusters, accompanied by a color change of the solution from gray back to red. More importantly, the previously formed **AB_2_** clusters at the pH of 10.0 mainly transited to stable **AB_3_** after this cycle of pH variation (Figure 3a; Figure S14, Supporting Information). This finding aligns with the changes observed in the UV/Vis spectra, specifically the declining in the intensity of the transverse plasmon band and the red‐shift of the longitudinal plasmon band (Figure S15, Supporting Information). When the pH was decreased from 10.0 to 3.0, the hydrodynamic diameters (*D*_h_) increased from 34.9 ± 0.7 nm to 230.1 ± 40.7 nm. Upon resuming the pH to 10.0, the *D*_h_ decreased to 46.5 ± 0.6 nm. The results indicate that the pH‐induced assembly and disassembly processes indeed occurred in the solution. When the pH value decreased from 11.0 to 3.0, NP‐**A**s exhibited no significant structural or surface charge alterations, maintaining a nearly constant zeta potential of +18 mV and a *D*_h_ of ≈65 nm (Figure 3c,d). In contrast, NP‐**B**s exhibited pronounced pH‐dependent behavior: their zeta potential shifted from −39 to −6 mV, accompanied by a marked reduction in *D*_h_ from 62.3 ± 0.7 nm to 27.9 ± 1.8 nm. We presume that this phenomenon originates from the pH‐responsive protonation of carboxylate groups (‐COO⁻) on PAA ligands. At lower pH values, protonation of −COO⁻ to −COOH groups reduced the negative charge density on NP‐**B**s, thereby weakening interparticle electrostatic repulsion. Concurrently, the decreased hydrophilicity of protonated PAA ligands induced chain collapse, as evidenced by the *D*_h_ reduction, which further diminished steric repulsion between NP‐**B**s. These dual effects—reduced electrostatic and steric stabilization—collectively enable the observed structural reorganization of NP‐**B** assemblies under acidic conditions. Furthermore, control experiments demonstrated that NP‐**A**s alone exhibited no pH‐responsive behavior, confirming that NP‐**B**s serve as the primary functional component responsible for the observed pH‐dependent cycling (Figure S16, Supporting Information). We, therefore, proposed a PAA ligand conformation‐dictated assembly mechanism as illustrated in Figure 3e. Under acidic conditions (pH 3.0), collapsed PAA ligands reduce both steric hindrance and electrostatic repulsion, enabling the recruiting of free NP‐**B**s by clusters. When the system is restored to alkaline conditions (pH 10.0), electrostatic repulsion between charged ligands stabilizes the newly formed clusters with an increased coordination number in solution.

**Figure 4:**
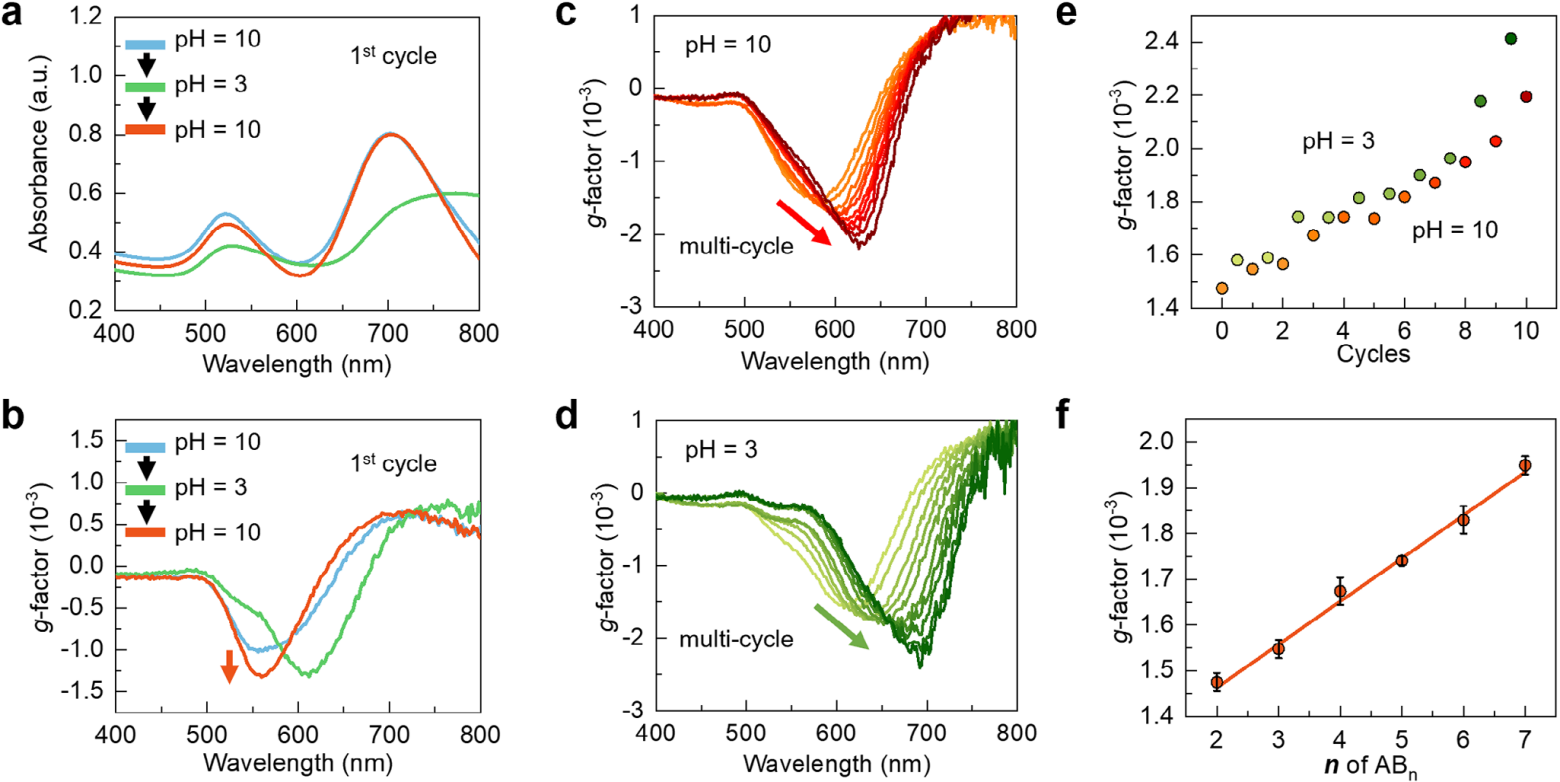
Optical activity of the NP assemblies during the base‐acid cycles. a) UV/Vis and b) *g*‐factor spectra of the NP assemblies during the first pH cycle. c,d) Evolution of *g*‐factor spectra after 10 cycles. e) *g*‐factor values over ten cycles. f) *g*‐factor values as a function of ***n*** in **AB_n_** clusters.

The pH‐induced changes in the structure of NP assembly also affect its optical activity (**Figure** **4**a,b). The absorption peak observed in the UV/Vis spectra at 520 nm was primarily attributed to the transverse plasmon band of NP‐**A**s and the free NP‐**B**s in solution. The intensity of this peak diminished at pH 3.0, aligning with the gradual reduction in free NP‐**B**s. The second absorption peak at 700 nm resulted from the longitudinal plasmon band of NP‐**A**s. Due to the significant NP aggregation at pH 3.0, this peak broadened and exhibited a noticeable redshift. Similarly, the CD peak underwent a redshift from 560 to 612 nm as the pH declined from 10.0 to 3.0. In order to eliminate the effect of UV/Vis spectra variations on CD intensity, the observed CD signal was quantified using the dissymmetry factor (*g*‐factor), which denotes the ratio of CD intensity to the corresponding absorption (see the Methods section). Notably, compared with the initially formed **AB_2_** clusters at pH 10.0, the *g*‐factor of the **AB_3_** slightly increased after the pH cycle (highlighted by the red arrow in Figure 4b).

The increased intensity of the CD signal was also demonstrated in further pH cycling experiments. To clearly illustrate the trend in *g*‐factor, the *g*‐factor spectra of NP assemblies at pH 3.0 and 10.0 were individually depicted in Figure 4c,d, respectively. Interestingly, the *g*‐factor values show consistent increments at both pH 3.0 and 10.0 as the number of pH cycles increased, with the *g*‐factor values enhanced by 1.7‐fold after 10 pH cycles. In addition, the peak wavelength of the CD signal undergoes a red‐shift with increasing pH cycles (Figure 4e). Given the enhanced aggregation by acid, the CD peak wavelength at pH 3.0 is always higher than that at pH 10.0. However, the *g*‐factor values at adjacent pH levels of 3.0 and 10.0 were very close and followed a similar trend as a function of the cycling number. This observation suggests that the increased *g*‐factor values during pH cycles primarily stem from the **AB_n_** clusters formed by the absorbed NP‐**B**s on NP‐**A**s rather than from the disordered **A_m_B_n_** aggregates. This also explains the phenomenon that the size of the aggregates increases without a change in the *g*‐factor. (Figure S17, Supporting Information). Remarkably, a linear rise in the *g*‐factor values across the ***n*** values of **AB_n_** was observed (Figure 4f). This is the first time that a linear correlation between inherent optical activity and the quantity of hotspots has been shown. In addition, the addition of trace NaCl after each pH cycle was found to have negligible effects on the structural integrity and chiroptical properties of chiral nanoparticle assemblies (Figures S18, S19, Supporting Information).

**Figure 5:**
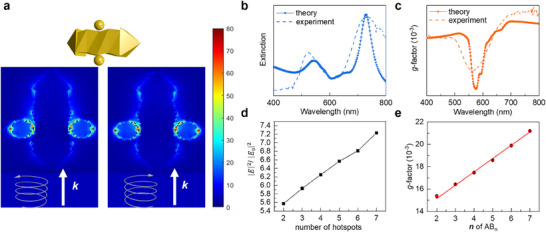
FDTD simulations of the chiroptical properties of the NP assemblies. a) The **AB_2_** model employed in the simulation and the electric‐field intensities on **AB_2_** upon normal incidence of LCP and RCP light. The light propagates in the direction marked with white arrows. b,c) Experimental and theoretical b) extinction and c) *g*‐factor spectra of **AB_2_** clusters. d) Simulated electric‐field intensities as a function of number of hotspots. e) Simulated *g*‐factor values as a function of ***n*** in **AB_n_** clusters.

In order to verify the linear relationship between the *g*‐factor and ***n*** value of **AB_n_** clusters, we carried out Finite‐Difference Time‐Domain (FDTD) simulations (**Figure** **5**). The simulated extinction, CD, and *g*‐factor spectra of **AB_n_** models show a good agreement with the corresponding experiment results, regarding their peak positions and handedness (Figure S20, Supporting Information). The simulated extinction spectra displayed an intense longitudinal LSPR peak at ≈720 nm and a relatively weak transverse LSPR peak at ≈540 nm (Figure S20a, Supporting Information), and their half‐width is narrower than experimental results due to the ideal simulated conditions. The CD and *g*‐factor spectra of NP‐**A**s models exhibited a negative peak at the transverse LSPR band. The FDTD simulation was carried out under ideal conditions and was not affected by the inhomogeneity of chiral gold nanoarrows structures. Thus, the simulated *g*‐factor spectrum is narrower than that of the experiment (Figure S20b, Supporting Information). The chiroptical signals of NP‐**A**s models also agreed with the twisted direction of the chiral nanoarrows side, further confirming that the NP‐**A**s models are consistent with the experiment results. We simulated the nearfield distribution of electric fields of the **AB_2_** model under irradiation of left circularly polarized (LCP) light and right circularly polarized (RCP) light at the LSPR band. The result showed that the electrical field is strongly localized in the joints of the NP‐**B**s and the NP‐**A**s in the **AB_2_** under both LCP and RCP (Figure 5a). This result confirmed that it is the coupling of NP‐**B**s and NP‐**A**s that affected the chiroptical response in the vis–near‐infrared region. Similarly, the simulated extinction and *g*‐factor spectra of the **AB_2_** model are in good agreement with the corresponding experimental results in terms of peak position and handedness (Figure 5b,c)

We simulated and calculated the electric field strength for different numbers of hotspots and found that the number of hotspots is linearly related to the electric field strength (Figure 5d; Figure S21, Supporting Information). Then, we simulated the chiroptical response in the vis–the near‐infrared region of **AB_3_**, **AB_4_**, **AB_5_**, **AB_6_**, and **AB_7_** models, the simulation results show a good agreement with the corresponding experiment results (Figure S20c–m; Figure S22, Supporting Information). In order to investigate the specific effect of hotspot location on the *g*‐factor, we simulated the chiral clusters of NP‐**B**s located on the top and side of NP‐**A**s, respectively (Figure S23, Supporting Information). The results show that compared to the hotspot formed on the top of the NP‐**A**s, the hotspot located on the side of the NP‐**A**s contributes more significantly to the system's optical chirality. Finally, we counted and fitted the simulated *g*‐factor separately, and we found that *g*‐factor has a linear relationship with ***n*** value of **AB_n_**, which is consistent with experimental results (Figure 5e). The results of the simulations confirm that the number of hotspots leads to a linear enhancement of the electric field strength thus leading to a linear relationship between the values of ***n*** value of **AB_n_** and *g*‐factor.

## 3 Conclusion

In summary, we have developed a highly effective approach for precisely controlling the self‐assembly of binary NPs, namely inherently chiral and achiral NPs, into a range of **AB_n_** clusters utilizing complementary polymers. The adjustment of polymer configurations induced by carboxy groups under varying pH conditions plays a pivotal role in precisely regulating the formation of self‐assembled **AB_n_** clusters on demand. Interestingly, the number of achiral NP‐**B**s within the assemblies demonstrated a notable impact on both the wavelength and intensity of the CD signals. Experimental and simulation data collectively establish a linear correlation between the inherent optical activity (quantified by the dissymmetry *g*‐factor) and the number of plasmonic hotspots within the clusters. This quantitative relationship arises from the enhanced local electric fields at interparticle junctions, which amplify chiroptical responses proportionally with an increasing number of plasmonic hotspots. Our findings establish a versatile platform for designing chiral plasmonic materials with programmable optical asymmetry for applications in ultrasensitive chiral sensing, adaptive optical systems, and quantum photonics.

## Conflict of Interest

The authors declare no conflict of interest.

## Supporting information



Supporting Information

## Data Availability

The data that support the findings of this study are available from the corresponding author upon reasonable request.
