# Supplementary material for: Polymer‐Driven Co‐Assembly of Achiral and Chiral Nanoparticles into Plasmonic Nanoclusters with Quantitatively Modulated Optical Chirality
Source: Adv Sci (Weinh). 2025 Jun 19;12(34):e04850. doi: 10.1002/advs.202504850 (PMC12442589; doi:10.1002/advs.202504850)
Supplement: Supplementary file 1 — Supporting Information [file ADVS-12-e04850-s001.docx]

Supporting Information

**Polymer-driven Co-assembly of Achiral and Chiral Nanoparticles into Plasmonic Nanoclusters with Quantitatively Modulated Optical Chirality**

Chongyang Yao^‡^, Huibin He^‡^, Weijia Kong, Liangyu Hu, Xiaoxue Shen, Jing Tao, Yutao Sang * and Zhihong Nie*

**Table of Contents**

[Experimental Procedures 2](#_Toc192609372)

[**1.Materials** 2](#_Toc192609373)

[**2. Synthesis of thiol-terminated poly (acrylic acid) (PAA-SH)** 2](#_Toc192609374)

[**3. Synthesis of spherical gold nanoparticles (Au NPs)** 2](#_Toc192609375)

[**4. Synthesis of helically grooved gold nanoarrows (*NP-As*)** 3](#_Toc192609376)

[***4.1 Synthesis of gold nanoarrows (GNRs)*** 3](#_Toc192609377)

[***4.2 Synthesis of helically grooved gold nanoarrows (NP-As)*** 3](#_Toc192609378)

[**5. Functionalization of** NP-**A**s **with thiol-terminated polyethylene glycol (PEG-SH) and HS(CH_2_)_11_NMe^3+^Br^-^ (TMA)** 3](#_Toc192609379)

[**6. Functionalization of Au NPs with thiol-terminated poly (acrylic acid) (PAA-SH)** 3](#_Toc192609380)

[**7. Self-assembly of NP-A and NP-B.** 4](#_Toc192609381)

[**8. Calculation of the *g*-factor.** 4](#_Toc192609382)

[**9. Simulation and calculation of electric field strength.** 4](#_Toc192609383)

[**10. Characterization** 4](#_Toc192609384)

[Supplementary Figures 4](#_Toc192609385)

[Reference 14](#_Toc192609386)

Experimental Procedures

**1.Materials**

4-cyano-4-(phenylcarbonothioylthio)pentanoic acid (CPPA), azo-bis(isobutyronitrile) (AIBN), n-butylamine, gold (III) chloride trihydrate (HAuCl_4_· 3H2O, ≥ 99.9% trace metals basis), sodium citrate, _L_-ascorbic Acid (AA), silver nitrate (AgNO3), sodium borohydride (NaBH4), HS(CH_2_)_11_NMe^3+^Br^-^ (TMA) and hydrochloric acid (HCl, 37 wt. % in water) were purchased form Sigma-Aldrich. Dioxane, tetrahydrofuran (THF), methanol, dichloromethane (DCM), *N,N*-dimethylformamide (DMF),ethyl acetate and trifluoroacetic acid (TFA) were purchased form Sinopharm Chemical Reagent Co., Ltd. Hexadecyltrimethylammonium bromide (CTAB), sodium oleate (NaOL), hexadecyltrimethylammonium chloride (CTAC) and *L*-Cystine (*L*-Cys) were purchased from TCI. Thiolated-PEG with different molecular weight (MW) were purchased from MeloPEG. AIBN was recrystallized twice from ethanol. *tert*-Butyl acrylate (*t*BA) was distilled under vacuum prior to use and stored in freezer at −18 ^o^C. All chemicals were used as received unless otherwise noted. Deionized water (Millipore Milli-Q grade) with resistivity of 18.0 MΩ was used in all the experiments.

**2. Synthesis of thiol-terminated poly (acrylic acid) (PAA-SH)**

The synthetic procedures of PAA-SH are illustrated in **Figure S3**. First, the PtBA-CTA copolymer was synthesized by using reversible addition fragmentation chain-transfer (RAFT) polymerization. *t*BA (7.69 g, 60 mmol), CPPA (56 mg, 0.20 mmol) and AIBN (4 mg, 0.025 mmol) were added to dioxane (5 mL) in a 25 mL Schlenk flask. The mixture was degassed by three cycles of freeze-pump-thaw. Then the flask was placed in an oil bath pre-heated to 70 ^o^C and the mixture under magnetic stirring for 24 hrs. In order to terminate the polymerization, the solution was quickly cooled down to room temperature by placing the flask in liquid nitrogen. The mixture was diluted with THF and then precipitated in the mixture of absolute methanol/water (v/v = 1:1) three times. The red precipitates were dried under vacuum at 50 ^o^C overnight. The yield was estimated to be ~50%.

In order to release the thiol end-group, 2.5 g P*t*BA-CTA were dissolved in 20 mL THF, and the mixture was stirred under N_2_ atmosphere for 30 min. Then n-butylamine (6.4 mmol, 0.85 mL) was added to the mixture under magnetic stirring. The reaction proceeded was stirred for 3 hrs under N_2_ atmosphere at room temperature. After the reaction, the product was purified by precipitating it in the mixture of absolute methanol/water (v/v = 1:1) and re-dissolving it in THF for three cycles. Then the resulting polymer was dried under vacuum at 50 ^o^C overnight. The *M_n, GPC_* of P*t*BA-SH was 24.1 Kg·mol^-1^ with *Đ* of 1.19. corresponding to the polymer structure of P*t*BA_188_-SH.

For the synthesis of PAA-SH, 2.0 g P*t*BA-SH were dissolved in 40 mL DCM, and the mixture was stirred under N_2_ atmosphere for 30 min. Then 10 mL TFA was added to the mixture, and the reaction was stirred at room temperature for 24 hrs. Because of the poor solubility of PAA in DCM, the hydrolytic polymer gradually precipitates out of the solution during hydrolysis. Excess DCM was removed by spin evaporation. In order to remove excess TFA, the product was dissolved in methanol and precipitated in ethyl acetate three times. Then the polymer was dried under vacuum at 50 ^o^C overnight to obtain PAA_188_-SH (Mn = 13.5 kDa).

**3. Synthesis of spherical gold nanoparticles (Au NPs)**

Au nanoparticles synthesized by using methods reported previously.^[1]^ To synthesize 15 nm-diameter Au NPs, 0.24 mL of HAuCl_4_·3H_2_O aqueous solution (50 mg·mL^-1^) were injected into 150 mL of boiling water under stirring. 4.5 mL of sodium citrate aqueous solution (10 mg·mL^-1^) was injected into the solution quickly. Then the mixture was refluxed for 30 min and cooled down to room temperature. The solution was cooled down to room temperature. In order to obtain larger Au NPs, the 15 nm-diameter Au NPs were used as seeds at 90 ^o^C. Another 4.5 mL of sodium citrate aqueous solution (10 mg·mL^-1^) and 0.24 mL of HAuCl_4_·3H_2_O aqueous solution (50 mg·mL^-1^) were sequentially injected into the mixture. The mixture was heated to 100 ^o^C under stirring. Then the reaction mixture was refluxed for 30 min and cooled down to 90 ^o^C. Repeat the growth procedure once, the 20 nm-diameter Au NPs were obtained. AuNPs with a diameter of 30 nm could be synthesized by four growth processes. **Figure S2** shows the TEM images and size distribution of the synthesized Au NPs.

**4. Synthesis of helically** **grooved gold nanoarrows (*NP-As*)**

NP-**A**s were obtained according to a seed-mediated method as reported previously.^[2]^ NP-**A**s with chiroptical properties could be synthesized by adding L- or D-Cysteine to the growth solution of grooved gold nanoarrows.

***4.1 Synthesis of gold nanoarrows (GNRs)***

GNRs were synthesized by a seed-mediated method reported previously.^[3]^ First, the seed solution for GNR growth was prepared as follows:2.5 mL of 0.5 mM HAuCl_4_ was added to 2.5 mL of 0.2 M CTAB solution under stirring. 0.5 mL of freshly prepared ice-cold 6 mM NaBH_4_ as a reducing agent was injected to mixture under vigorous stirring (1200 rpm). After 2 min of vigorous stirring, the seed solution was aged at 30 ^o^C for 30 min before use. Second, to prepare the growth solution, 3.5 g of CTAB and 617 mg of NaOL were dissolved in 250 mL of 50 ^o^C water in a 500 mL Erlenmeyer flask. The solution was cooled down to 30 ^o^C and 9 mL of 4 mM AgNO_3_ was added. Then the 125 mL of 1 mM HAuCl_4_ solution was added and the mixture was kept undisturbed at 30 °C for 15 min. After 90 min of stirring, the solution became colorless. The 1.05 mL HCl (37 wt. % in water, 12.1 M) was added. After 15 min of stirring, 0.625 mL of 0.064 M ascorbic acid (AA) was injected and vigorously stirred for 30 s. Finally, 1.2 mL of seed solution was introduced into the growth solution. The resultant mixture was stirred for 30 s and kept undisturbed at 30 ^o^C for 12 h for GNRs growth. GNRs were centrifuged twice (6000 rpm, 10 min) to remove unreacted reagents, concentrated 10-fold in volume and then re-dispersed in a 1 mM CTAB solution as the seeds for NP-**A**s growth.

***4.2 Synthesis of helically grooved gold nanoarrows (NP-As)***

A growth solution of NP-**A**s was prepared by adding 3.6 mL of 10 mM HAuCl_4_ into 120 mL of 100 mM CTAC under stirring.1.8 mL of 10 mM AgNO_3_ and 2.4 mL of 1 M HCl were added into the mixture sequentially. Subsequently, 1.8 mL of 0.1 M ascorbic acid (AA) was injected and the solution was vigorously stirred for 30 s. Followed by the addition of 240 μL *L*-Cys (5 μM in the final growth solution) and 6 mL of GNRs seeds solution. After undisturbed placing at 30 °C for 4 h, NP-**A**s could be obtained when the solution changed from red to brown. The final products were isolated by centrifugation at 5,000 rpm for 10 min, concentrated 10-fold in volume, and then re-dissolved into aqueous CTAB solution (1 mM) for storage.

**5. Functionalization of** NP-**A**s **with thiol-terminated polyethylene glycol (PEG-SH) and HS(CH_2_)_11_NMe^3+^Br^-^ (TMA)**

The surface of NP-**A**s were functionalized by using two-step ligand exchange method. Briefly, the solution of NP-**A**s was centrifuged twice (4500 rpm, 5 min) to remove excess CTAB and was re-dispersed in water. Then 1 mL aqueous PEG solution was dropwise added into 1 mL of NP-**A**s in a 4 mL glass vial under sonication. Subsequently, the mixture was kept sonicated for 30min and then vortexed overnight to ensure complete ligand exchange. After centrifugation twice (5000 rpm, 10 min) to remove the untethered polymer, the PEG-functionalized NP-**A**s was re-dispersed in water. Then, 0.2 mL of aqueous TMA solution was dropwise added into 2 mL of PEG-modified NP-**A**s under vortex. After vortexing overnight, the PEG and TMA functionalized NP-**A**s were isolated by 2 cycles of centrifugation (4500 rpm, 10 min). Finally, the NP-**A**s were re-dispersed in water as an assembly unit **A** for future use. The concentrations of NP-**A**s, PEG and TMA mentioned above are showed in Supporting Table 1.

**6. Functionalization of Au NPs with thiol-terminated poly (acrylic acid) (PAA-SH)**

The PAA-SH was grafted onto the surface of the Au NPs by ligand exchange method. The citrate-stabilized AuNPs were isolated by centrifugation followed by removal of the supernatant, concentrated 10-fold in volume, and then re-dispersed into DMF. Then 1 mL PAA dissolved in DMF was dropwise added into 1 mL of AuNPs under sonication. The mixture was kept sonicated for 30min and then vortexed overnight to allow complete ligand exchange. The PAA-modified Au NPs were purified to remove unreacted PAA-SH by 4 cycles of centrifugation. Then the Au NPs were re-dispersed in 1 mL of water as an assembly unit **B** for future use. The concentrations of Au NPs and PAA are listed in Supporting Table 1.

**7. Self-assembly of NP-A and NP-B.**

The NP-**B**s solution are injected into a specific volume of NP-**A**s solution in a 10:1 particle ratio under sonication, and the mixture was sonicated for 30 seconds to ensure uniform mixing. Then the assembly cluster was obtained. By adding HCl (0.1 M) and NaOH (0.1 M) to the solution to control the pH of the assembly, different assembly clusters can be obtained, and the corresponding chirality can be changed. Following the addition of the HCl or NaOH solution, the mixture was rapidly mixed using a pipette before proceeding with UV-vis, DLS, CD and TEM measurements.

**8. Calculation of the *g*-factor.**

The asymmetry factor of the polarization rotation, also known as the *g*-factor, is a concentration independent parameter.^[4]^ It is calculated as the ratio of the different absorbances of a substance between LCP light and RCP light to the total absorbance using the following equation

$$g \mathrm{factor}=\frac{A_{LCP}-A_{RCP}}{A_{LCP}+A_{RCP}}=\frac{\Delta A}{A_{\mathrm{total}}}\approx\frac{CD(m deg)}{32980\times abs}$$

**9. Simulation and calculation of electric field strength.**

For the **AB_2_**, **AB_3_** and **AB_4_** models, only the electric field strength in the XZ plane needs to be simulated. And as the number of hotspots increases, it is also necessary to simulate the electric field strength in the YZ plane on top of that. Figure S15 illustrates the simulation schematic for the **AB_2_**-**AB_7_** models. The simulation results are shown in Table 3. From the obtained results, we can learn that the addition of new hotspots does not affect the electric field strength of the original hotspots, and the number of hotspots is linearly related to the electric field strength. Based on the above two points, we deduce the electric field strengths of the **AB_5_**, **AB_6_** and **AB_7_** models.

**10. Characterization**

***Gel Permeation Chromatography (GPC).*** GPC experiments were performed on an Agilent/Wyatt GPC 1260 equipped with a UV detector. HPLC-grade DMF containing 0.1 M LiBr was used as an elution solvent at a flow rate of 1.0 mL·min^-1^. PMMA standards were used for molecular weight and molecular weight distribution calibration.

***UV-visiable Absorption Spectroscopy.*** UV-vis spectra were measured on a Perkin-Elmer Lambda.

***TEM imaging.*** TEM imaging was performed using a Hitachi HT7800 High Contrast Transmission Electron Microscope (HC-TEM). TEM samples were prepared by casting 2 µL of sample solution on 300 mesh copper grids covered with carbon film, and drying them at room temperature. All the TEM grids were treated by air plasma using a plasma cleaner (PDC 32G-2, Harrick) for 60 seconds prior to use.

***Dynamic Light Scattering (DLS) and Zeta Potential experiments.*** DLS experiments and zeta potential experiments were carried out on a Zetasizer-Nano 90 from Malvern at 20 °C, equipped with a He-Ne 633 nm laser as the light source.

***Circular dichroism (CD).*** Circular dichroism (CD) spectra were obtained on a Chirascan Series Spectrometer (Applied Photo Physics Ltd, UK) at a scan range of 400 to 800 nm, 0.5 s time per point, 1-nm step size, and a 1-nm bandwidth.

***SEM imaging*.** The NP-**A**s were characterized using a Zeiss S55 field emission gun Scanning Electron Microscope (FEG-SEM). To prepare samples for SEM imaging, 1 μL of the sample solution was dropped onto a hydrophilic silicon substrate which was treated with piranha solution. The substrate with the sample solution was then allowed to dry at room temperature before conducting the SEM measurement.

Supplementary Figures


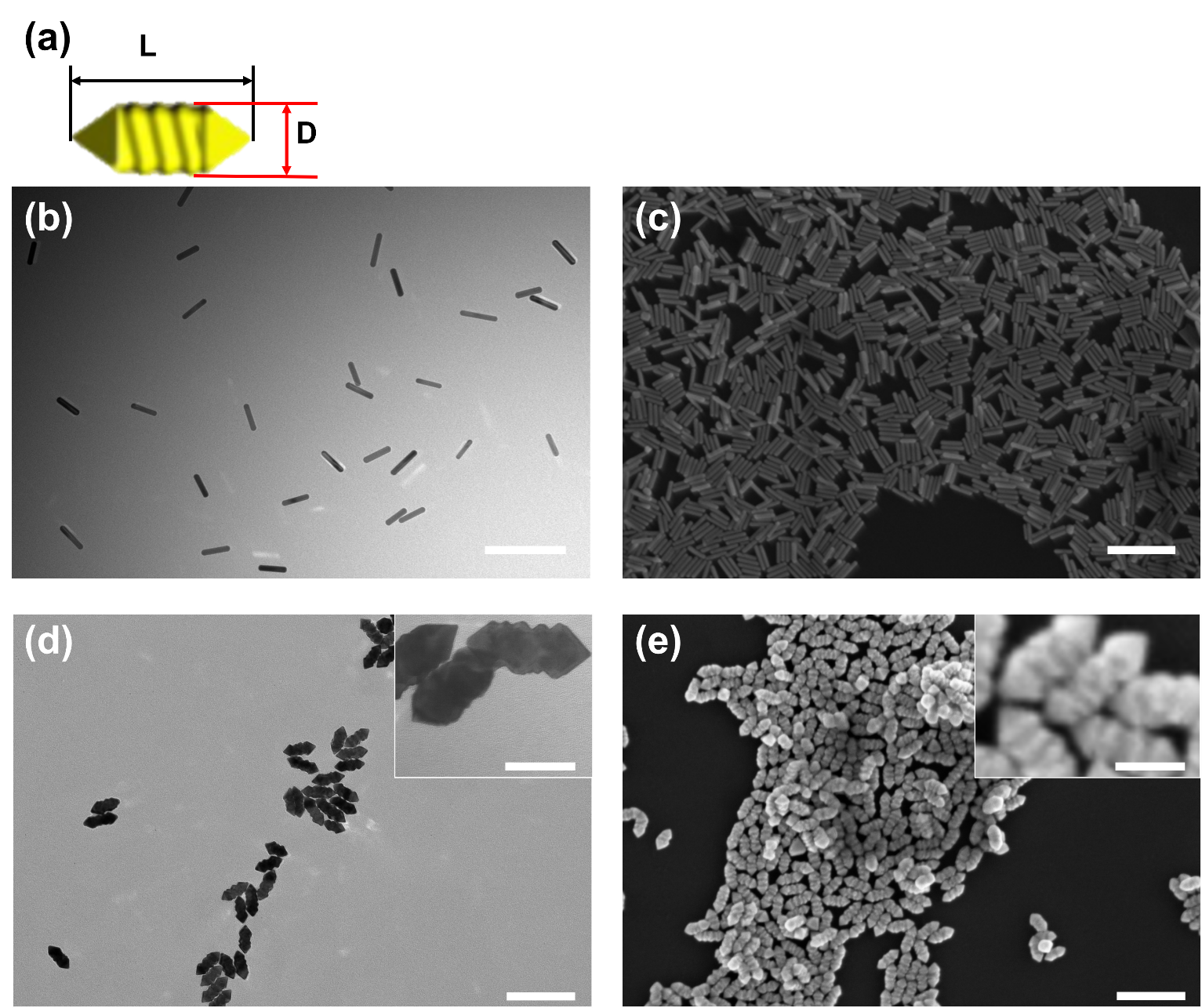


Figure. S1. (a) Definition of geometric parameters of NP-As. (b-e) TEM and SEM images of synthesized (b, c) GNRs (L=69.9 ± 3.3 nm, D=13.5 ± 0.7 nm) and (d, e) NP-As (L=87.1± 2.6 nm, D=37.3 ± 0.8 nm). Scale bars are 200 nm and inset scale bars are 50 nm.


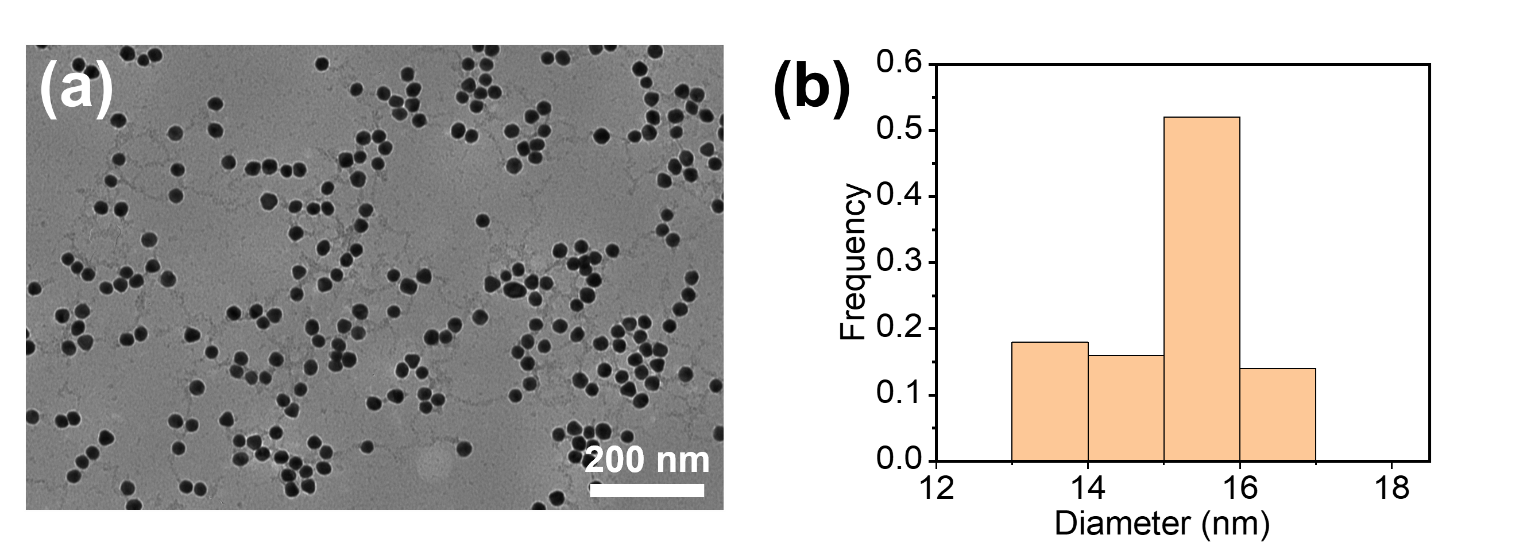


**Figure S2.** (a) TEM images and (b) size-distribution histograms of synthesized Au NPs (15.1 ± 1.2 nm).


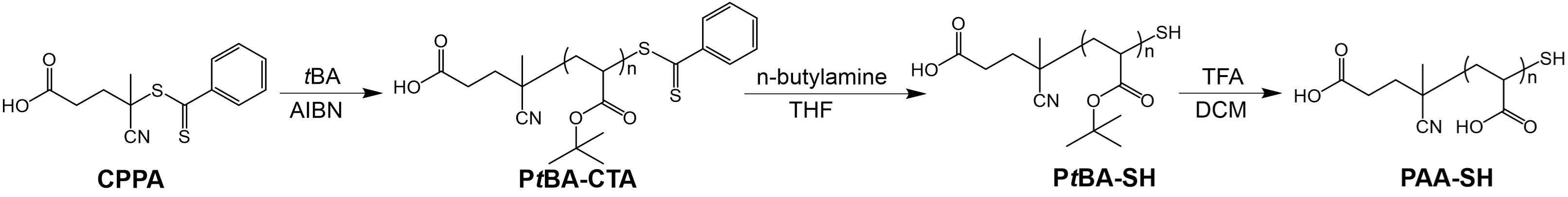


**Figure S3.** The synthetic routes of PAA-SH.

**Table S1**. The ligand exchange conditions of representative nanoparticles.

| NPs | *C*_NP_^a^ (mg/ml) | *C*_Polymers_^b^ (mg/mL) | *C*_TMA_^c^ (μg/mL) |
| --- | --- | --- | --- |
| ***A***@PEG20k/TMA | 0.18 | 0.53 | 10 |
| ***B***-15@PAA13.5k | 1.44 | 4.0 |  |

Note: a) The concentration of AuNPs in the reaction mixture during ligand exchange. b) The concentration of PAA or PEG in the reaction mixture during ligand exchange. c) The concentration of TMA in the reaction mixture during ligand exchange.


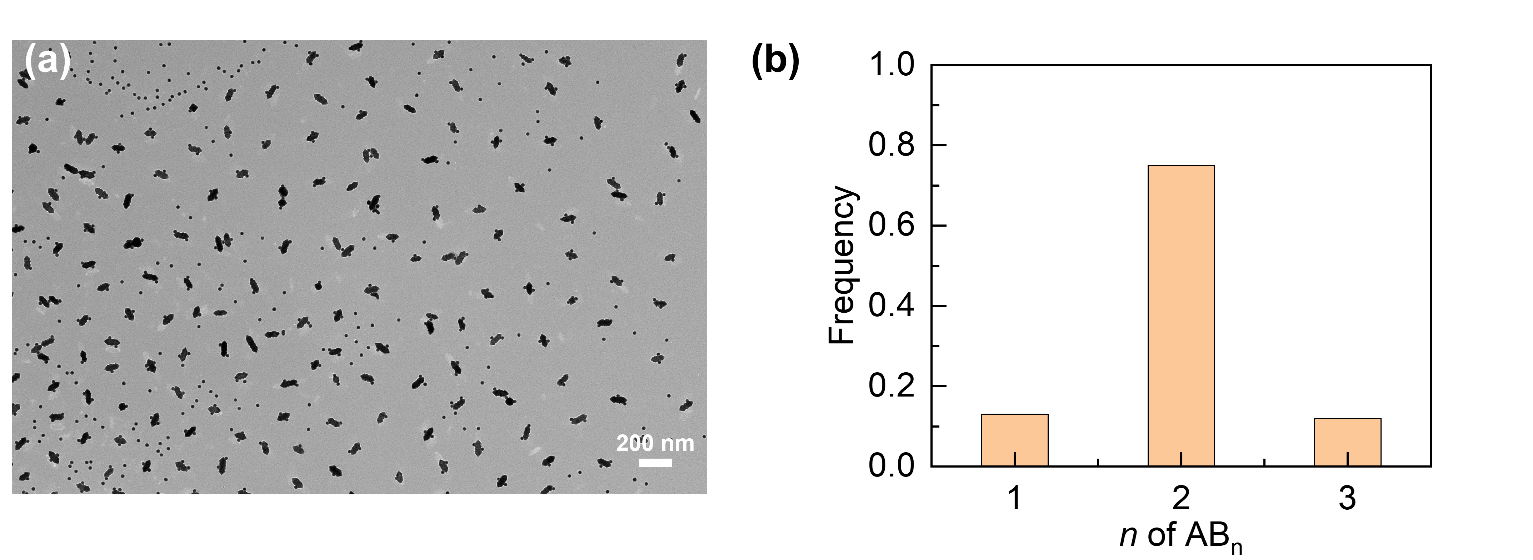
**Figure S4.** (a) TEM images of assembly cluster at pH = 10.0 and (b) corresponding calculated the yield of **AB_n_** cluster (***n*** is the number of NP-**B**s).


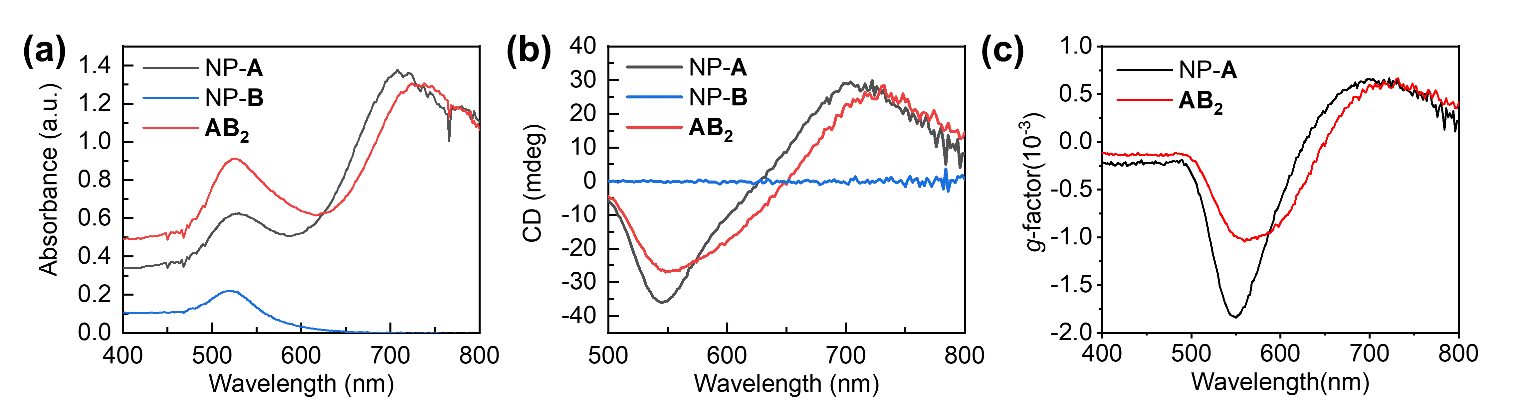


**Figure S5.** (a) UV-visible absorption spectra, (b) CD and (c) corresponding calculated *g*-factor of NP-**A**s, NP-**B**s and the **AB_2_** cluster.


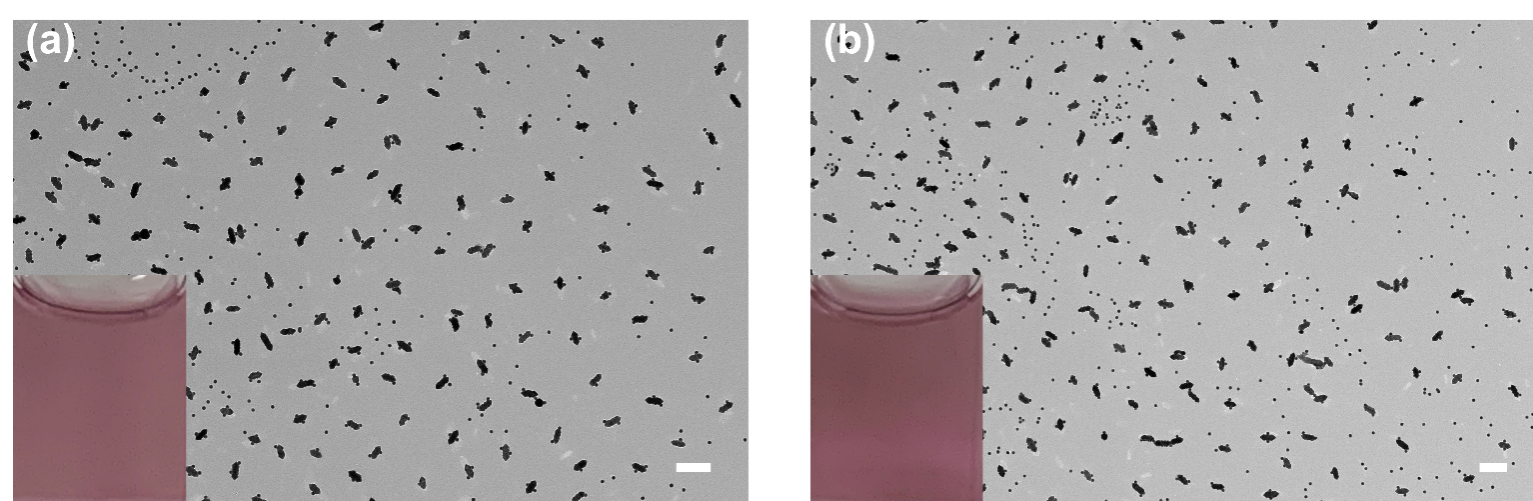


**Figure S6.** TEM images and photos of **AB_2_** cluster (a) right now and (b) after 30 days. Scale bars are 200 nm.


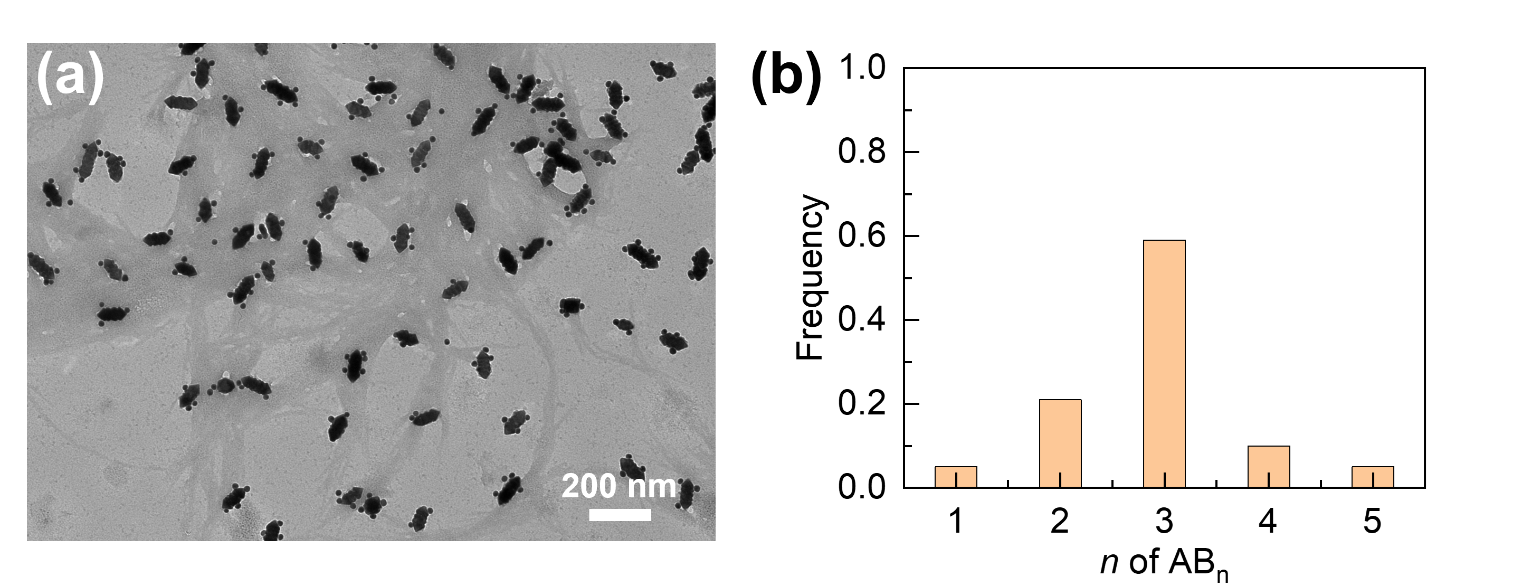
**Figure S7.** (a) TEM images of assembly cluster at pH = 10.0 after one cycle and (b) corresponding calculated the yield of **AB_n_** cluster (***n*** is the number of NP-**B**s).


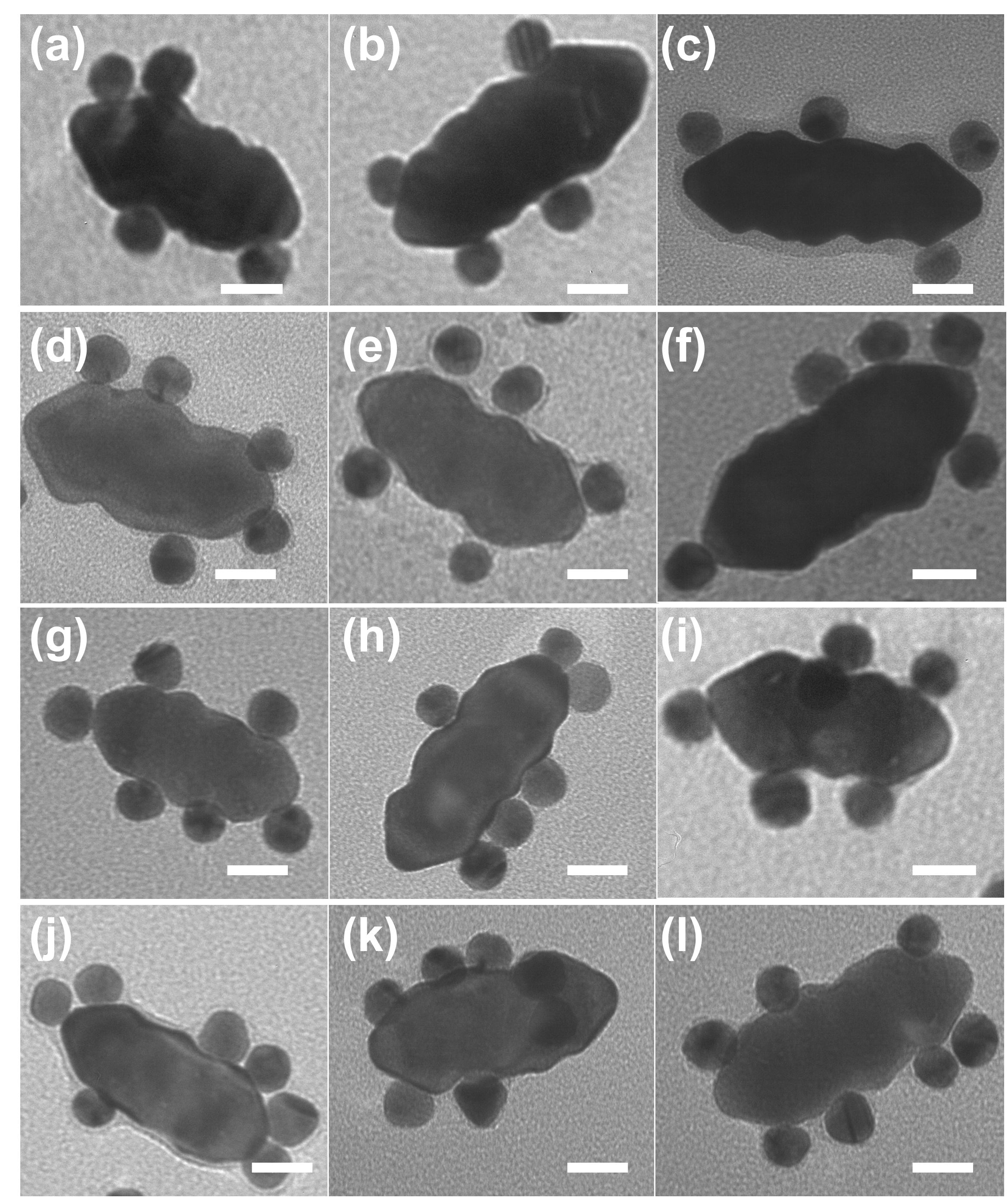


**Figure S8.** The high contrast TEM images of (a-c) **AB_4_**, (d-f) **AB_5_**, (g-i) **AB_6_** and (j-l) **AB_7_** clusters. Scale bars are 20 nm.


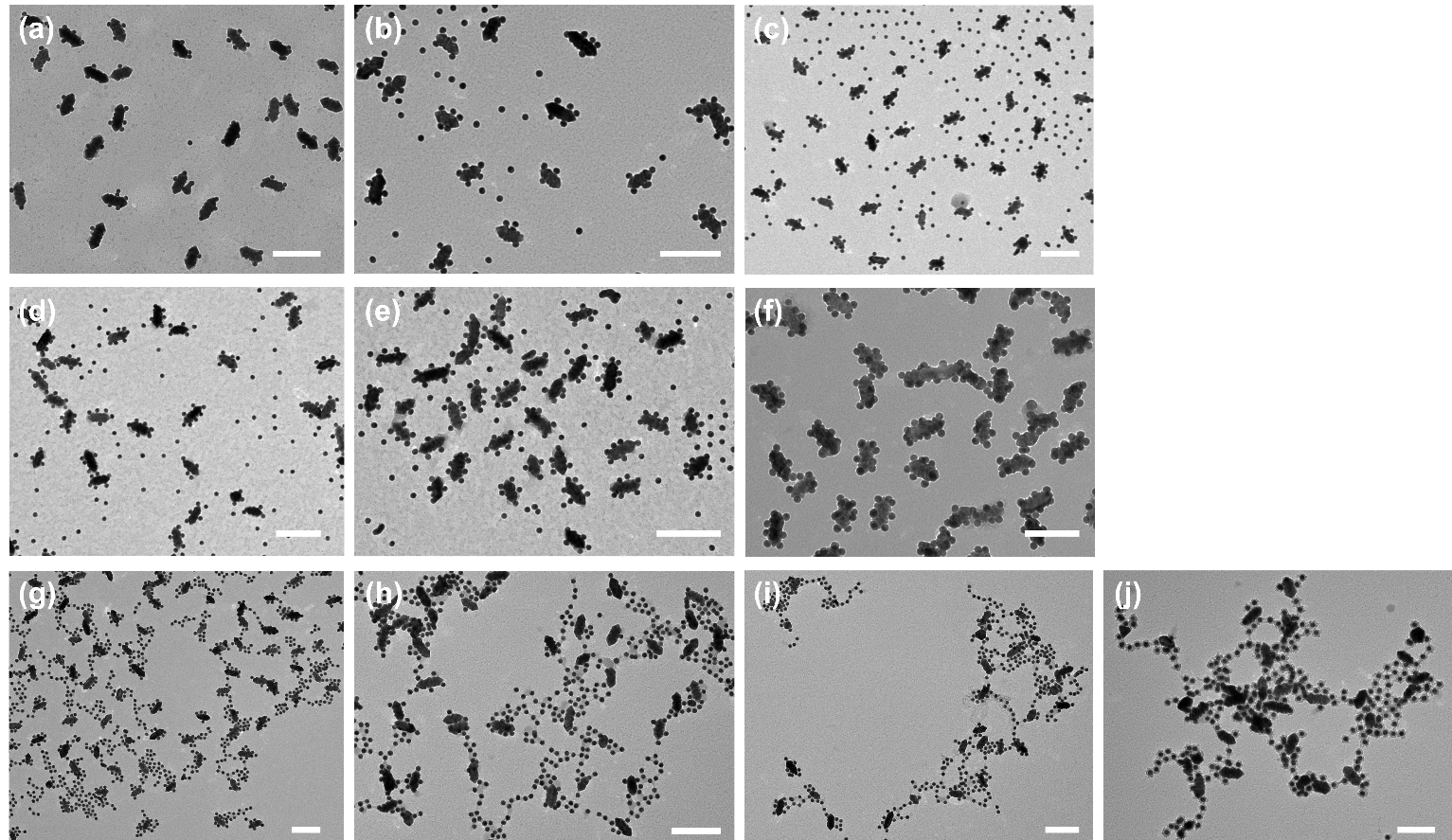


**Figure S9.** (a-j) TEM images of the assembly with 10 cycles at pH = 10.0. Scale bars are 200 nm.

**Table S2**. The **AB_n_** cluster at pH = 10.0 in first 6 cycles. ***n*** was counted three times and averaged into‾n corresponding to results in Figure S8.

| Cycles | *n*_1_ | *n*_2_ | *n*_3_ | ‾n |
| --- | --- | --- | --- | --- |
| **1** | 2.88 | 2.84 | 2.94 | 2.89 |
| **2** | 3.96 | 4.08 | 4.02 | 4.01 |
| **3** | 5.05 | 4.95 | 5.07 | 5.02 |
| **4** | 6.03 | 6.16 | 6.19 | 6.13 |
| **5** | 6.24 | 6.16 | 6.35 | 6.25 |
| **6** | 6.78 | 6.95 | 6.85 | 6.86 |

Note: More than 100 assembly cluster were counted each time.


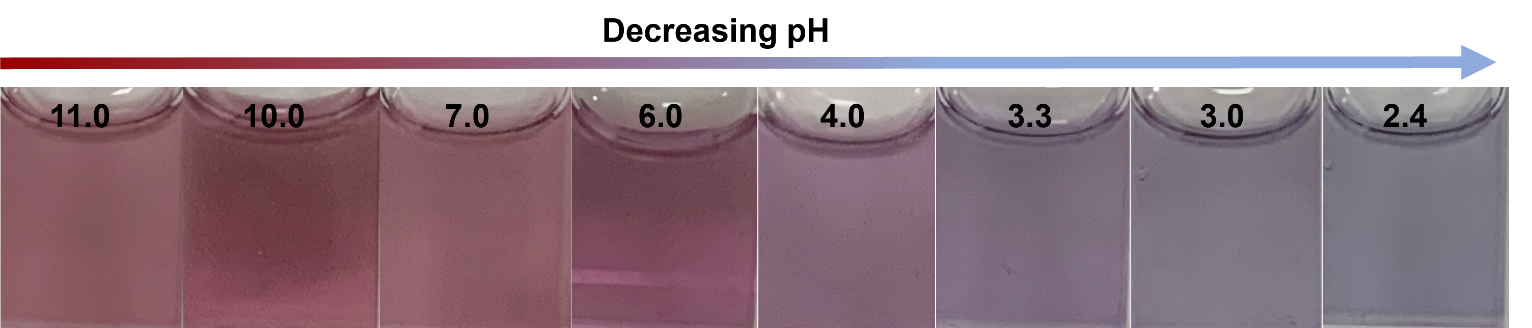


**Figure S10.** Photos of the solution of **AB_2_** cluster under different pH values.


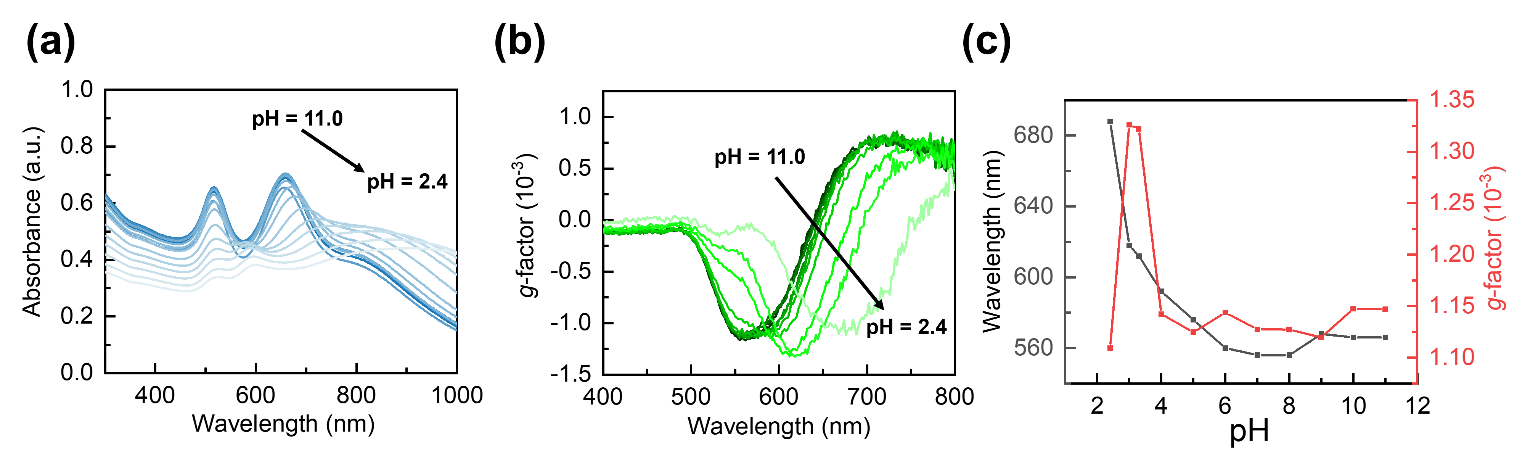


**Figure S11.** pH-dependent (a) UV-vis spectra and (b) *g*-factor of the assembled clusters. (c) The dependence of SPR band and *g*-factor on pH.


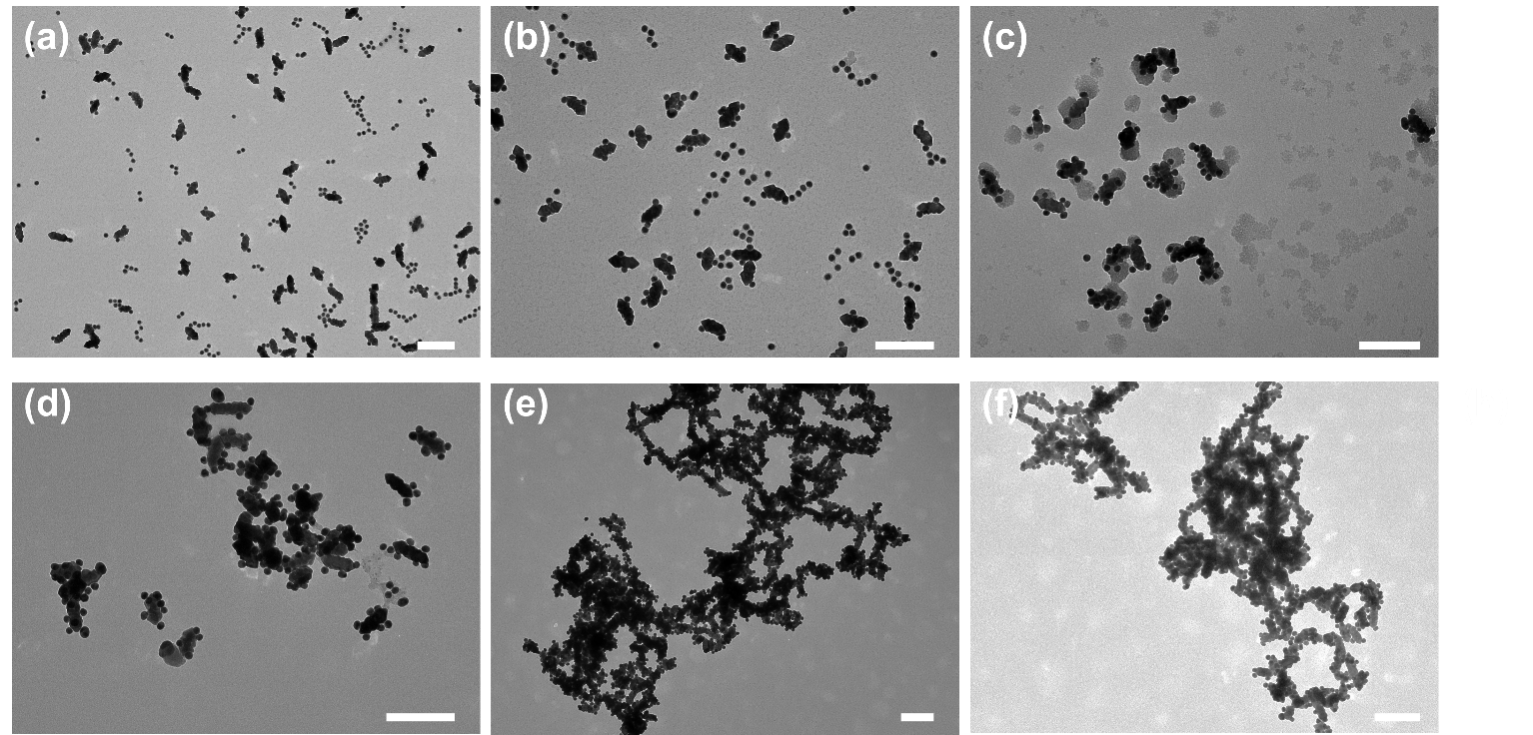


**Figure S12.** TEM images of pH-dependent assembly. (a) pH = 7.0 (b) pH = 6.0 (c) pH = 3.3 (d) pH = 3.0 (e) pH = 2.7 (f) pH = 2.4. Scale bars are 200 nm.


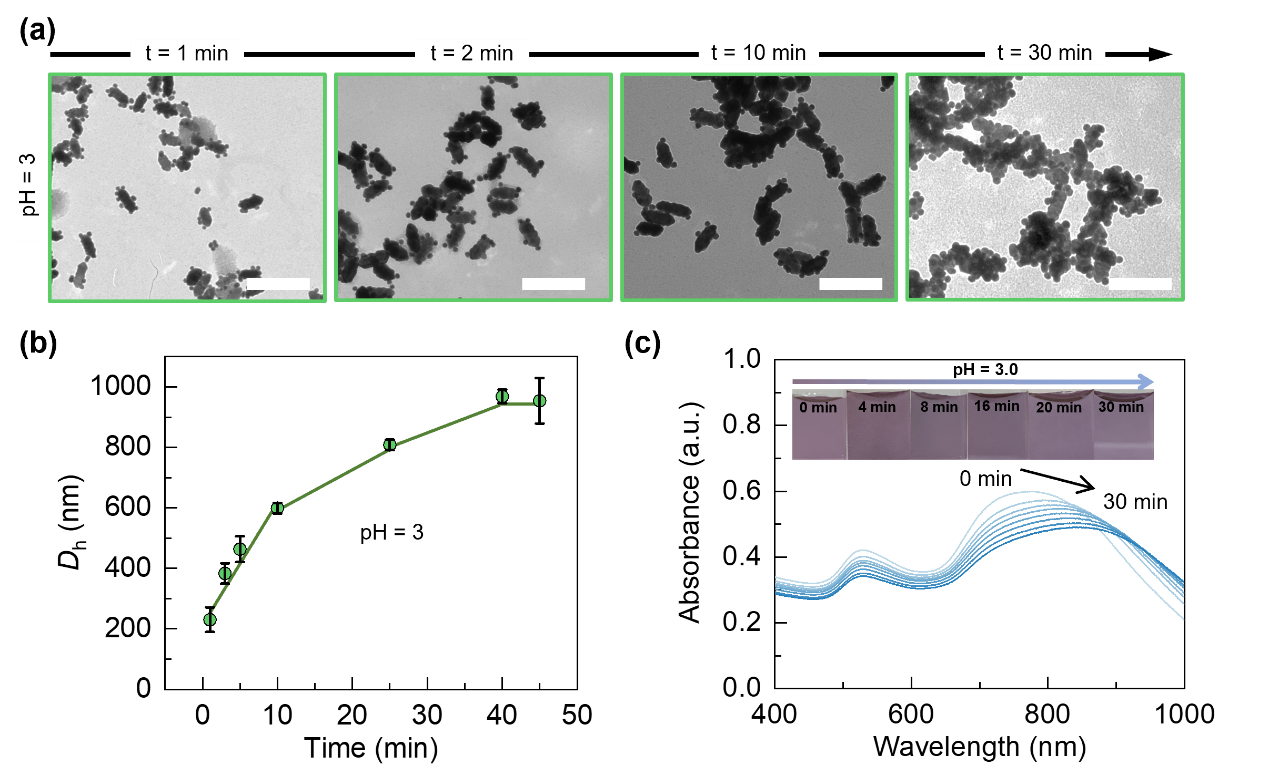


**Figure S13.** (a) TEM images, (b) *D*_h_ and (c) UV/Vis of time-dependent NPs self-assembly at pH 3.0. Insets in (c) are photos of the corresponding NP solution under different time. Scale bars are 200 nm.


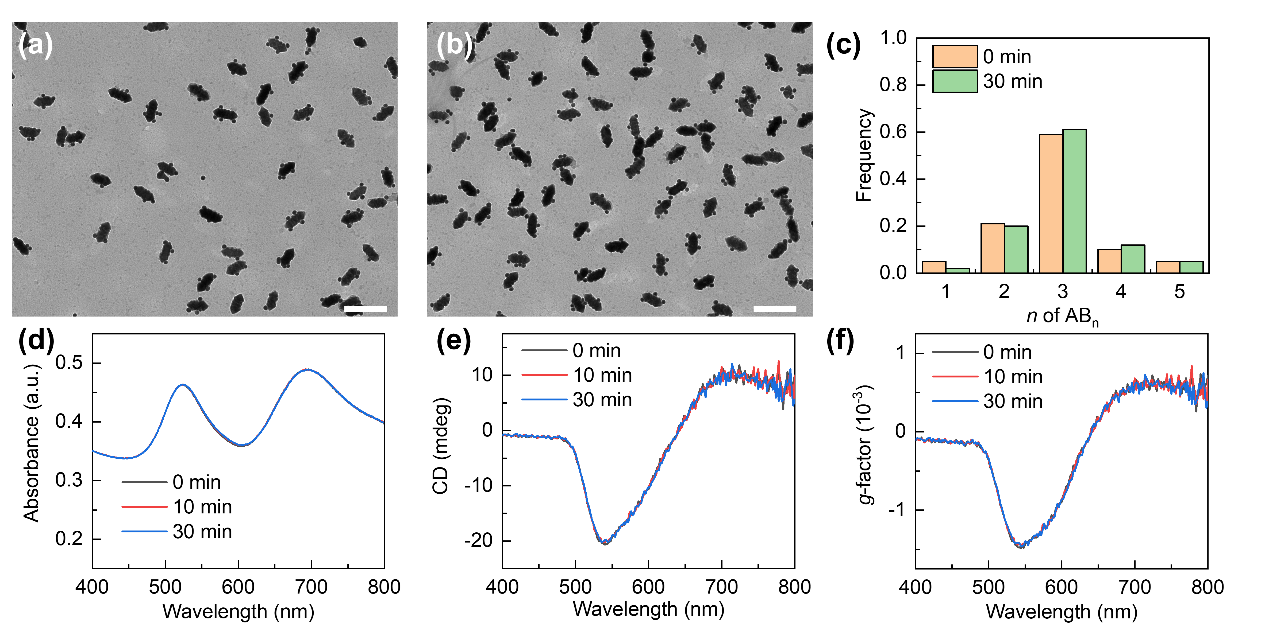


**Figure S14.** TEM images of **AB_3_** clusters prepared (a) immediately and (b) after 30 min. (c) Yield distribution of **AB_n_** cluster corresponding to results in (a) and (b). (d) UV/Vis, (e) CD and (f) *g*-factor of **AB_3_** clusters at pH 10.0. Scale bars are 200 nm.


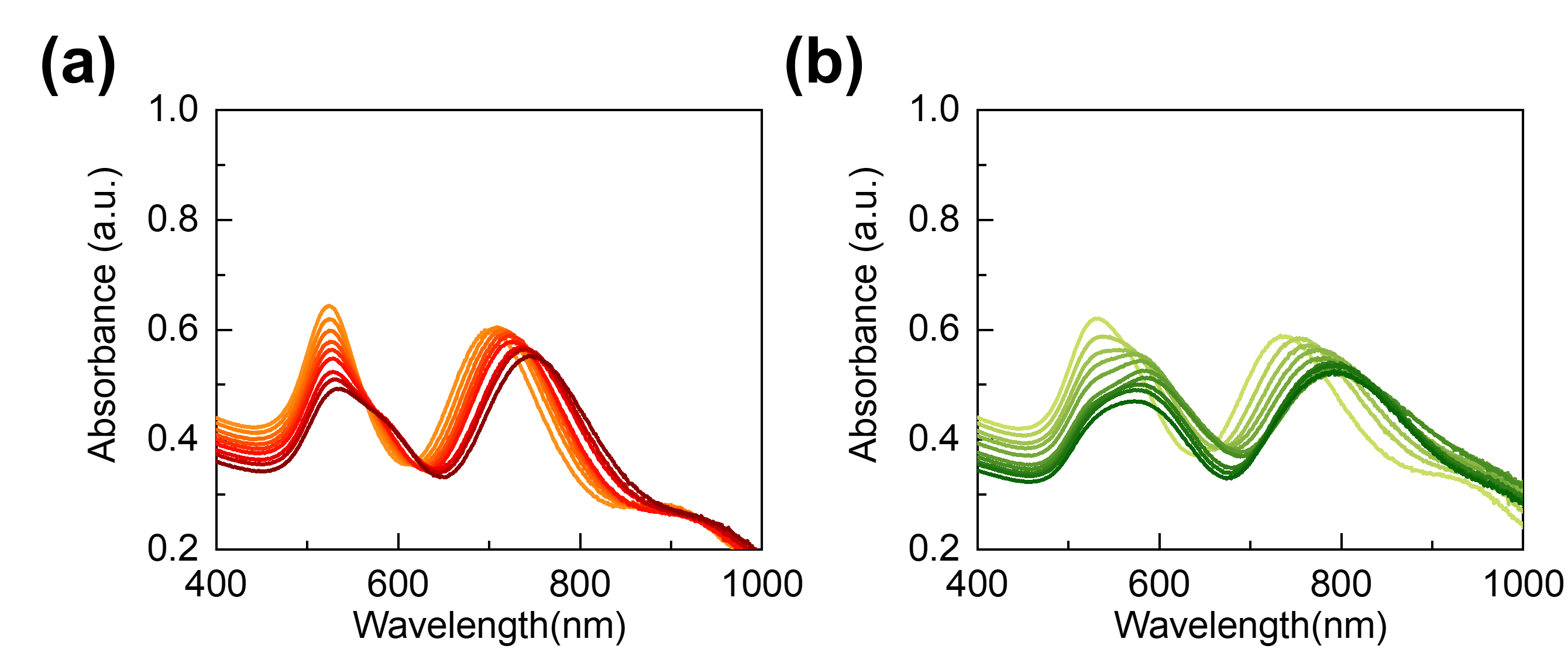


**Figure S15.** UV/Vis spectra of the NP clusters in 10 cycles, (a) pH = 10.0, (b) pH = 3.0.


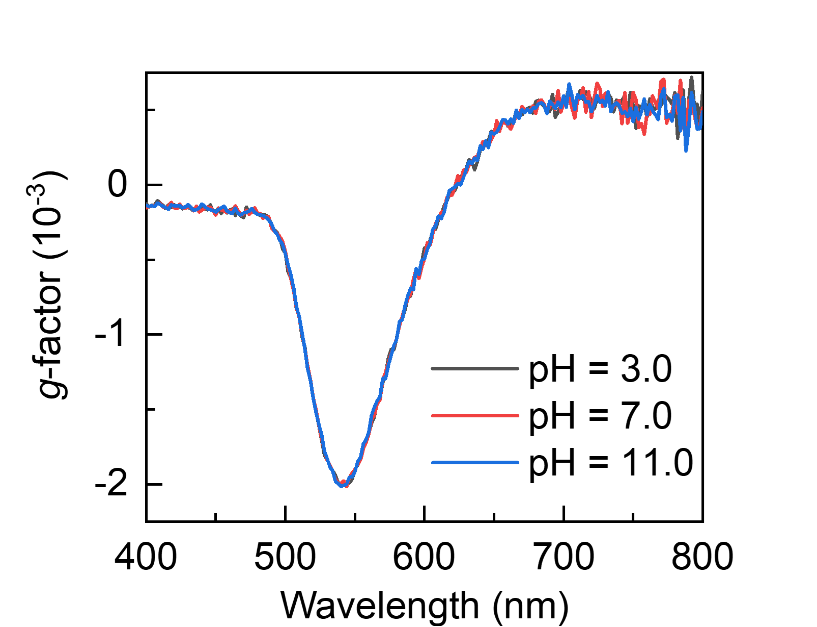


**Figure S16.** The *g*-factor spectra of NP-**A**s under different pH values.


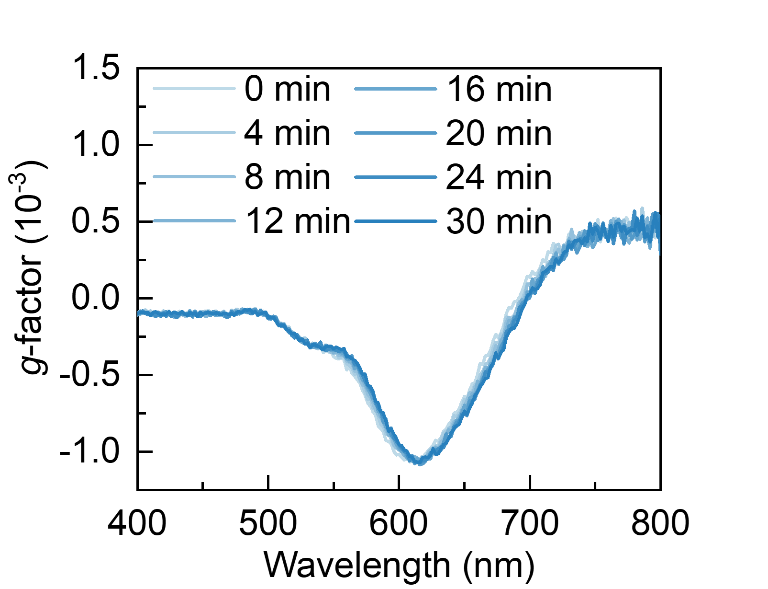


**Figure S17**. Time-dependent *g*-factor spectra of NP assemblies at pH = 3.0.


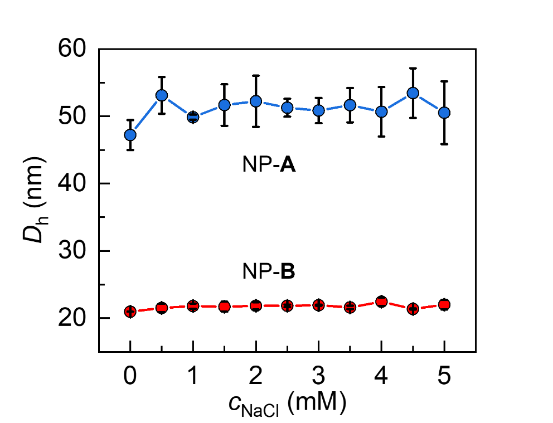


**Figure S18.** Hydrodynamic diameters (*D*_h_) of NPs at different *c*_NaCl_. The results show remarkable stability of both nanoparticle systems under varying ionic conditions.


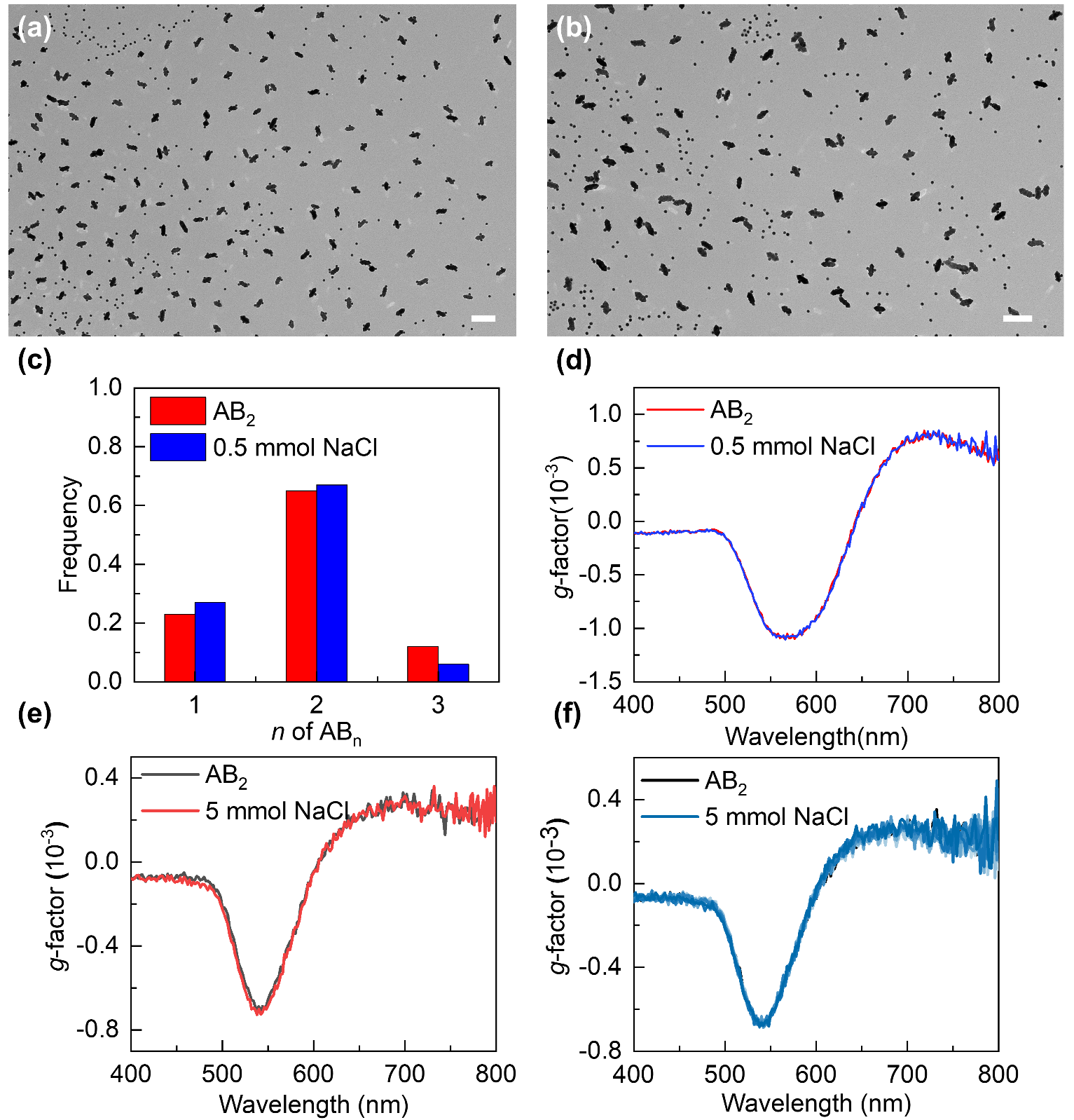


**Figure S19.** TEM images of (a) **AB_2_** and (b) **AB_2_** + 0.5 mmol NaCl. Scale bars are 200 nm. (c) The yield of **AB_n_** cluster (***n*** is the number of NP-**B**s) corresponding to results in Figure S14 a and b. (d) *g*-factor of **AB_2_** and **AB_2_** + 0.5 mmol NaCl. (e-f) *g*-factor of **AB_2_** and **AB_2_** + 5 mmol NaCl (e) One-time addition, (f) gradual addition.


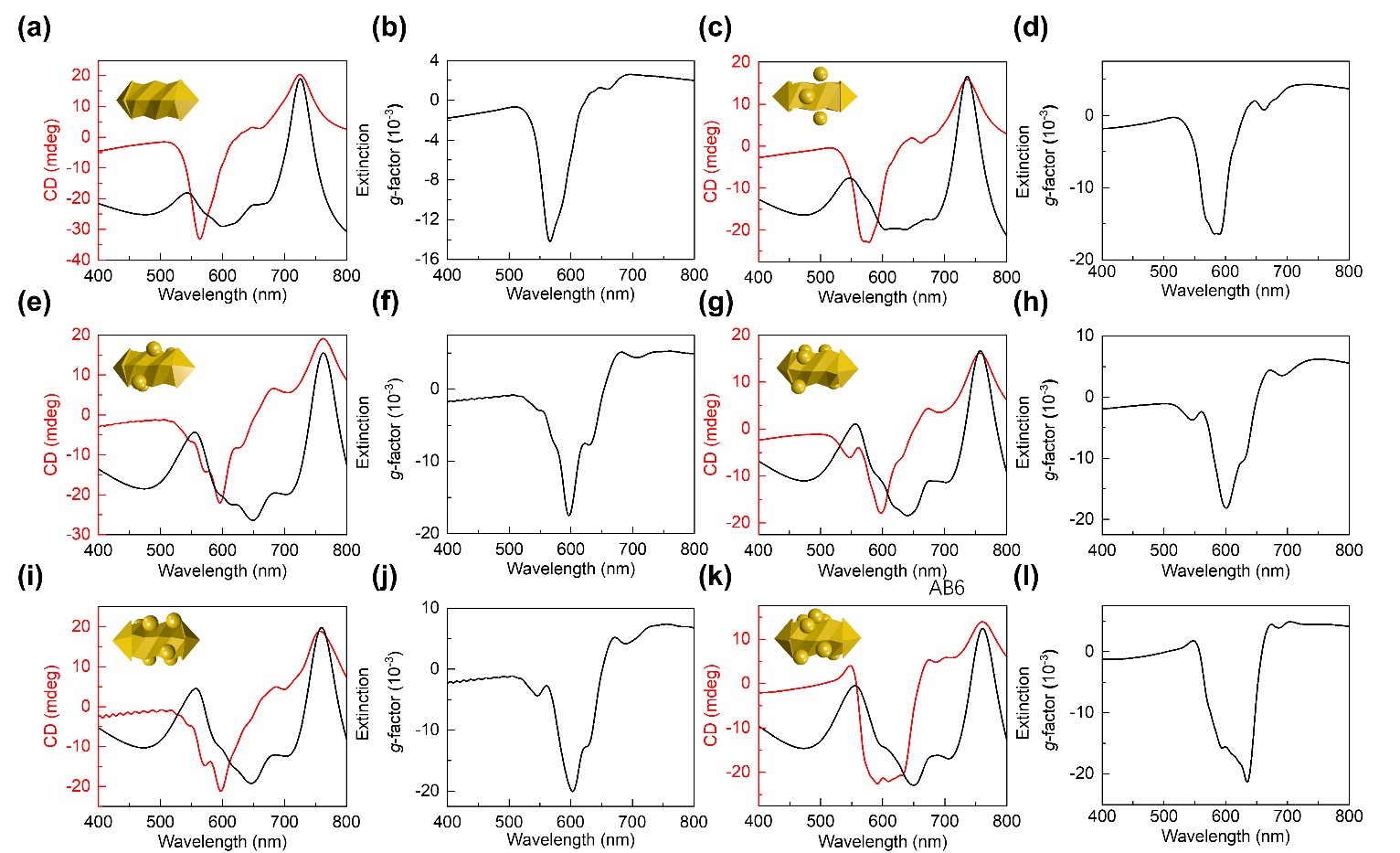
**Figure S20.** FDTD simulation of the chiroptical properties of NP-**A**s and chiral cluster. (a-b) NP-**A**s, (a) circular dichroism, extinction spectra and (b) *g*-factor. (c-d) **AB_3_**, (c) circular dichroism, extinction spectra and (d) *g*-factor. (e-f) **AB_4_**, (e) circular dichroism, extinction spectra and (f) *g*-factor. (g-h) **AB_5_**, (g) circular dichroism, extinction spectra and (h) *g*-factor. (i-j) **AB_6_**, (i) circular dichroism, extinction spectra and (j) *g*-factor. (k-l) **AB_7_**, (k) circular dichroism, extinction spectra and (l) *g*-factor. Inset are the NP-**A**s and chiral cluster models used in simulation.


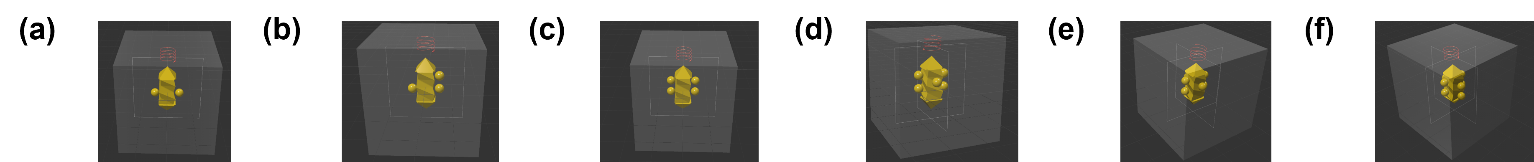


**Figure S21.** Schematic electric field simulation of (a) **AB_2_**, (b) **AB_3_**, (c) **AB_4_**, (d) **AB_5_**, (e) **AB_6_** and (f) **AB_7_** models.

**Table S3**. The electric field strength of the **AB_n_** models obtained from the calculation of Figure S14.

|  | **AB_2_** | **AB_3_** | **AB_4_** | **AB_5_** | **AB_6_** | **AB_7_** |
| --- | --- | --- | --- | --- | --- | --- |
| **XZ** | 5.572 | 5.9288 | 6.2516 | 6.2847 | 6.2347 | 6.2493 |
| **YZ** |  |  |  | 5.1789 | 5.5236 | 5.993 |
| **Total** | 5.572 | 5.9288 | 6.2516 | 6.5637 | 6.813 | 7.2308 |

Note: The electric field strengths for models **AB_5_**, **AB_6_** and **AB_7_** were estimated from the results obtained.


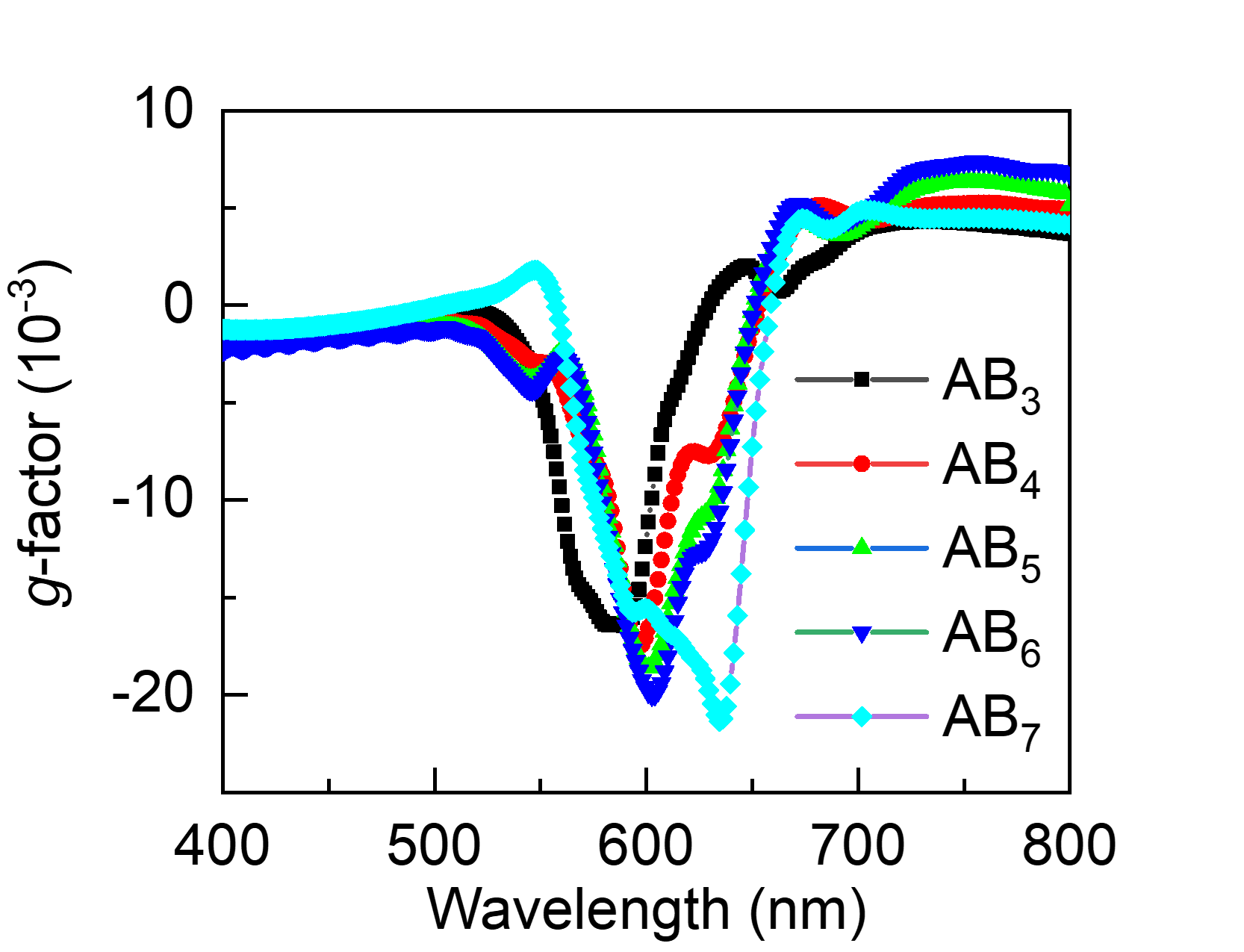


**Figure S22.** The simulated *g*-factor spectra of the chiral **AB_3_**, **AB_4_**, **AB_5_**, **AB_6_** and **AB_7_** clusters models.


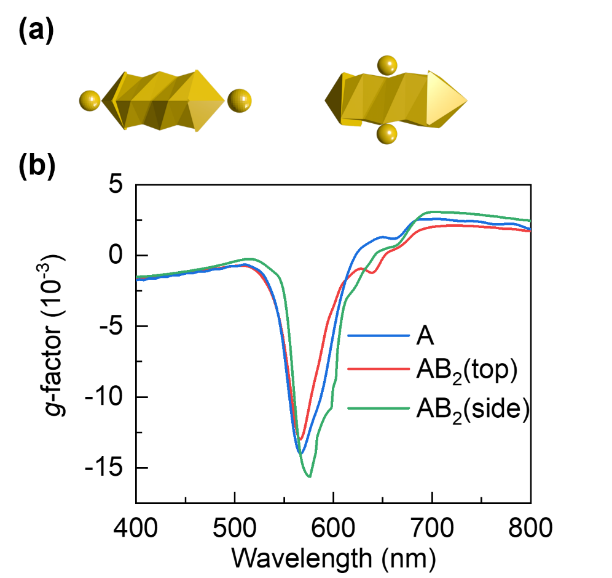


**Figure S23.** (a) Models of chiral **AB_2_** cluster with hot spots at different positions. (b) The simulated *g*-factor spectra of NP-**A**s and **AB_2_** cluster.

Reference

[1] H. He，X. Shen，C. Yao，J. Tao，W. Chen，Z. Nie，Y. Wu，L. Dai，Y. Sang, "Hierarchically Responsive Alternating Nano-Copolymers with Tailored Interparticle Bonds" *Angew. Chem. Int. Ed.* **2024,** *63*, e202401828.

[2] S. Wang，L. Zheng，W. Chen，L. Ji，L. Zhang，W. Lu，Z. Fang，F. Guo，L. Qi，M. Liu, "Helically Grooved Gold Nanoarrows: Controlled Fabrication, Superhelix, and Transcribed Chiroptical Switching" *CCS Chemistry* **2020,** *3*, 2473-2484.

[3] X. Ye，C. Zheng，J. Chen，Y. Gao，C. B. Murray, "Using Binary Surfactant Mixtures To Simultaneously Improve the Dimensional Tunability and Monodispersity in the Seeded Growth of Gold Nanorods" *Nano Lett.* **2013,** *13*, 765-771.

[4] J. Yan，W. Feng，J.-Y. Kim，J. Lu，P. Kumar，Z. Mu，X. Wu，X. Mao，N. A. Kotov, "Self-Assembly of Chiral Nanoparticles into Semiconductor Helices with Tunable near-Infrared Optical Activity" *Chem. Mater.* **2020,** *32*, 476-488.
